# The safety and efficacy of third- and fourth-generation cryoballoons for atrial fibrillation: a systematic review and meta-analysis

**DOI:** 10.3389/fcvm.2024.1364893

**Published:** 2024-08-12

**Authors:** Man-Li Zhang, Chao Zhang, Jian-Yong Peng, Shu-Qiao Xing, Jian Guo, Chen-Long Wei, Neng-Fang Zhang, En Ma, Wen-Sheng Chen

**Affiliations:** ^1^ Department of Cardiovascular Surgery, Xi’an Gaoxin Hospital, Xi’an, Shaanxi, China; ^2^ Department of Ultrasound, Xi’an Gaoxin Hospital, Xi’an, Shaanxi, China; ^3^ Department of Pharmacy, Hangzhou Normal University, Zhejiang, Hangzhou, China

**Keywords:** atrial fibrillation, third-generation cryoballoon, fourth-generation cryoballoon, safety and efficacy, meta-analysis

## Abstract

**Objectives:**

An increasing number of studies have shown that third (CB3)- and fourth-generation cryoballoons (CB4) have been used to treat various types of atrial fibrillation (AF), but previous research regarding the safety and efficacy of CB3 or CB4 ablation remains controversial. Therefore, a meta-analysis was performed to further evaluate the safety and efficacy of pulmonary vein isolation (PVI) using the CB3 and CB4 in the treatment of AF.

**Methods:**

We searched PubMed, the Cochrane Library, Web of Science, China National Knowledge Infrastructure, Wanfang, China Science and Technology Journal Database, and Clinicaltrials.gov up to December 2023 for qualified trials and data extraction according to inclusion and exclusion criteria. All analyses were carried out using Review Manager 5.3 software.

**Results:**

The meta-analysis included 13 observational studies consisting of 3,281 subjects and did not include a randomized controlled trial. Overall analyses indicated that the CB3 significantly reduced total procedure time [weighted mean difference (WMD) = −8.69 min, 95% confidence interval (CI) = −15.45 to −1.94 min, I^2^ = 93%], increased the PVI recording [relative risk (RR) = 1.24, 95% CI = 1.03–1.49, I^2^ = 90%], and increased the mean nadir temperature of overall PVs (WMD = 2.80°C, 95% CI = 1.08–4.51°C, I^2^ = 89%) compared with the CB2. Moreover, the CB4 significantly reduced the total procedure time (WMD = −14.50 min, 95% CI = −20.89 to −8.11 min, I^2^ = 95%), reduced the fluoroscopy time (WMD = −2.37 min, 95% CI = −4.28 to −0.46 min, I^2^ = 95%), increased the PVI recording (RR = 1.40, 95% CI = 1.15–1.71, I^2^ = 90%) compared with the CB2. Time-to-isolation, the success rate of PVI, AF recurrence, and complications in the CB3 and CB4 were not significantly different compared with the CB2.

**Conclusion:**

These findings demonstrated that the CB3 and CB4 tended to be more effective than the CB2 in the treatment of AF, with shorter procedure times, more PVI recording, and similar safety endpoints.

## 1 Introduction

Atrial fibrillation (AF) is the most common type of arrhythmia, which is characterized by a rapid and irregular heart rate and loss of effective systolic function (1). The incidence and prevalence of AF have increased steadily over the past two decades, and it is estimated to affect more than 30 million people worldwide (2). The increased incidence of AF is strongly associated with hypertension, diabetes, coronary artery disease, and congenital heart disease. The wider use of more sensitive methods of rhythm monitoring has allowed the earlier detection of arrhythmia (3). In addition, the Framingham Heart Study indicated that AF increases the risk of developing stroke, heart failure, and all-cause mortality in the community, and was related to impaired cognitive function and longitudinal cognitive decline (4). Therefore, the research and development of new drugs and treatments for AF are still needed.

With the maturation and widespread use of cardiac electrophysiology, pulmonary vein isolation (PVI) has proven to be an effective measure in the treatment of AF, and it is still the cornerstone of catheter ablation for all types of AF. At present, the guidelines recommend that patients with symptomatic paroxysmal or persistent AF who are refractory to or intolerant of anti-arrhythmic medications should be treated with catheter ablation (5). Catheter ablation significantly decreases the risk of death, stroke, and hospitalization compared with anti-arrhythmic drugs or rate control drug therapy alone (6). Catheter ablation is a minimally invasive intervention that includes radiofrequency ablation, cryoballoon ablation (CBA), and pulsed field ablation. A previous study showed that the first-generation and second-generation cryoballoons significantly reduced procedural time and major complications compared with radiofrequency ablation (7). Furthermore, patients treated with the second-generation cryoballoon (CB2; Arctic Front Advance, Medtronic, Minneapolis, MN, USA) had less procedure time, less fluoroscopic time, and fewer incidences of AF recrudescence, as well as fewer complications compared with the first-generation cryoballoon (8). With the rapid development of CBA, third-generation cryoballoon (CB3; Arctic Front Advance ST, Medtronic, Minneapolis, MN, USA) and fourth-generation cryoballoon (CB4; Arctic Front Advance PRO, Medtronic, Minnesota, MN, USA) have been used in the clinical treatment of AF. Several centers have published their initial clinical experience with the CB3 or CB4 cryoablation system and compared them with the CB2. However, despite that, several studies that evaluated the safety or efficacy of CB3 or CB4 ablation, some of its indicators were still inconclusive. For instance, compared with the CB2, the CB3 better evaluates the time of PVI and shortens the fluoroscopy time and total procedure time (9). By contrast, several other investigations showed that the CB3 did not shorten the fluoroscopy time and total procedure time in AF ablation (10, 11). Furthermore, similar inconsistent conclusions were observed in previous studies comparing the safety and efficacy of the CB4 and CB2 (12, 13). Consequently, it is still necessary to comprehensively explore the impact of the efficacy and safety indexes in AF patients after CB3 or CB4 ablation. However, no such investigation has been conducted as far as we know.

Based on this background, we herein performed a meta-analysis that synthesized the results from all the available research to assess the safety and efficacy of CB3 and CB4 therapy for AF patients in the hope of providing a basis for the selection of patients and clinical application.

## 2 Methods

### 2.1 Literature search strategy

The systematic literature search was performed according to the Preferred Reporting Items for Systematic Reviews and Meta-Analyses (PRISMA) guidelines (Supplementary Table S1). Scientific databases (PubMed, Cochrane Library, Web of Science, China National Knowledge Infrastructure, Wanfang Database and China Science and Technology Journal Database, and Clinicaltrials.gov) were searched from database inception to December 2023 for eligible original studies comparing CB3 vs. CB2 or CB4 vs. CB2 therapy for AF. The search terms “fourth-generation cryoballoon,” “third-generation cryoballoon,” and “atrial fibrillation” were used in the search. No restrictions were applied to regions or languages. In addition, we manually searched the reference lists of all eligible original studies to include any missed relevant articles. The literature search was conducted by M-LZ and CZ independently, and detailed search information is featured in Supplementary Table S2.

### 2.2 Eligibility criteria

This study included randomized controlled trials (RCTs) and observational studies (cross-sectional, case–control, and cohort studies) that investigated the safety or efficacy of the CB3 or CB4 in comparison with the CB2 in patients with AF. Eligible studies were considered if they met the following PICO (population, intervention, comparison, and outcome) criteria: (1) the participants were AF patients, (2) the interventions were the CB3 or CB4, (3) comparators were the CB2, and (4) the qualified studies reported outcomes of at least one of the following regarding safety or efficacy: procedure time, total cryoablation time, fluoroscopy time, total freezing time, left atrial dwell time, success rates of PV, real-time PV isolations observed, total time-to-isolation (TTI), overall mean nadir temperature, AF recrudescence, pericardial effusion/cardiac tamponade, phrenic nerve palsy (PNP), groin complication, atrioesophageal fistula, symptomatic PV stenosis, and stroke/transient ischemic attack (complication). Animal trials, case reports, books and documents, laboratory studies, review articles, conferences, and abstracts were excluded in the present study.

### 2.3 Data extraction and study qualitative assessment

J-YP and S-QX independently extracted data from the qualifying studies and any different judgments were resolved by discussion among all researchers. Extracted data included the following information: the name of the first author, publication year, country, study design, detailed information about the generation of the cryoballoon, number of patients, gender, age, body mass index (BMI), left ventricular ejection fraction (LVEF), follow-up time, AF type, number of paroxysmal AFs, left atrium diameter (LAD), diabetes, hypertension, coronary artery disease, stroke/transient ischemic attack (medical history), and above outcomes of safety or efficacy. Incomplete data were not included in the meta-analysis. Meta-analysis was only performed if each type of data appeared more than three times in the eligible studies. The data describing the basic characteristics of the patient were summarized in a table, and the data on the treatment of AF with CBA were summarized for statistical analysis and displayed in the form of a figure.

Because of the observational design of the included studies, the Newcastle-Ottawa Quality Assessment Scale (NOS) was used to evaluate the quality of studies. In addition, the risk of bias in non-randomized studies was assessed by the NOS, a 0–9 star system. High-quality studies with an NOS score ≥5 were identified, and studies with an NOS score <5 were considered as low-quality studies. The NOS score was assessed by two independent researchers.

### 2.4 Statistical analysis

All statistical analyses were performed using Review Manager software, version 5.3. The 95% confidence interval (95% CI) of the risk ratio or mean difference was used to evaluate the safety and efficacy of CBA in the treatment of AF patients, and *P* ≤ 0.05 was considered statistically significant. As all included studies were observational designs, all analyses were conducted using a random-effects model. Continuous variables were expressed as mean ± standard deviation (SD), and categorical variables were expressed as *n* (%); therefore, the weighted mean difference (WMD) and relative risk (RR) were used to combine the effect quantity. Cochran's Q and I^2^ statistics were used to analyze the heterogeneity. An I^2^ value of 25% indicated low heterogeneity, 25%–50% indicated medium heterogeneity, and more than 50% indicated high heterogeneity. In addition, we performed sensitivity analysis to explore the sources of heterogeneity and stability of outcomes by successively deleting each study. Funnel plot analysis was used to assess the potential publication bias. The appearance of a symmetrical inverted funnel shape suggested there was no publication bias.

## 3 Results

### 3.1 Study characteristics and quality evaluation of the literature

**Figure 1:**
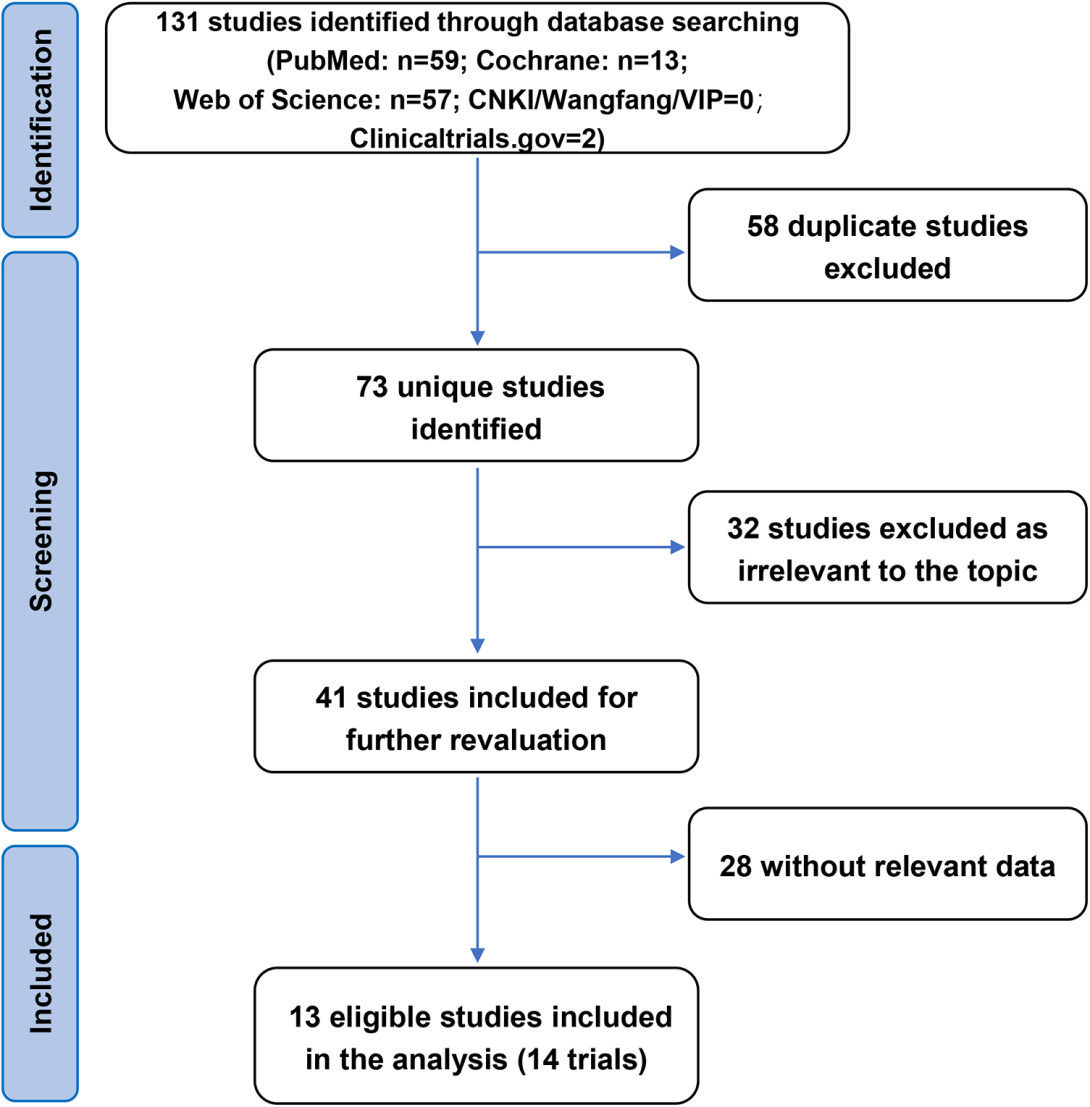
Flow diagram of the included studies. CNKI, China National Knowledge Infrastructure; VIP, China Science and Technology Journal Database.

A total of 131 records were identified from the six electronic databases. After excluding 58 articles that were duplicate studies, 32 articles that were not relevant to the purpose of this study were eliminated by title and abstract and 28 articles without relevant data were excluded; 13 articles were finally included in the meta-analysis study (9–21) (Figure 1). All 13 eligible articles were observational studies; no RCT was included. The characteristics of each included original analysis are shown in Table 1. The publications were from 2016 to 2022, most of which were conducted in Europe. Of the 3,281 patients in all the selected studies, 2,039 received the CB2 ablation procedure, 527 received the CB3 ablation procedure, and 715 received the CB4 ablation procedure. The mean age of the patients ranged from 55.5 to 66.8 years. All studies included paroxysmal AF and persistent AF patients and were independently assessed for quality using the NOS. The overall quality of the studies was rated as high quality.

**Table 1:** Baseline characteristics of the included trials.

Reference	Country	Study design	AF type	PAF	Comparison/intervention	Number of patients	Gender (male)	Age (y)	BMI (kg/m^2^)	LVEF (%)	LAD (mm)	Diabetes	Hypertension	CAD	Stroke/TIA	Follow-up time (months)	NOS
Aryana et al. (9)	USA	Cohort study	PAF or PerAF	254 (71.5%)	CB2/CB3	355 (253/102)	243 (68.5%)	64 ± 10.3	30.3 ± 6.0	55.3 ± 9.5	43.6 ± 6.1	82 (23.1%)	242 (68.1%)	74 (20.8%)	42 (11.8%)	12 ± 2	8
Fürnkranz et al. (14)	Germany	Cohort study	PAF or PerAF	380 (80.5%)	CB2/CB3	472 (423/49)	274 (58%)	64 ± 12	NA	NA	40 ± 6	48 (10%)	352 (75%)	75 (16%)	62 (13%)	NA	6
Heeger et al. (15)	Germany	Retrospective study	PAF or PerAF	42 (70%)	CB2/CB3	60 (30/30)	51 (85%)	61.3 ± 4.2	NA	NA	45 ± 6	4 (6.7%)	36 (60%)	7 (11.7%)	NA	NA	5
Heeger et al. (10)	Germany	Retrospective study	PAF or PerAF	71 (64.6%)	CB2/CB3	110 (55/55)	61 (55.5%)	62.1 ± 11.4	27.5 ± 4.8	NA	45.0 ± 6	12 (11%)	69 (63%)	12 (11%)	7 (6%)	12	5
Iacopino et al. (11)	Italy	Cohort study	PAF or PerAF	182 (75.8%)	CB2/CB3	240 (120/120）	171 (71.3%)	60.6 ± 10.6	26.9 ± 5.7	58 ± 6.7	41.5 ± 7.5	19 (7.9%)	105 (43.8%)	NA	9 (3.8%)	NA	7
			PAF or PerAF	172 (71.7%)	CB2/CB4	240 (120/120)	174 (72.5%)	61.4 ± 9.7	27.3 ± 4.6	57.9 ± 6.7	40.2 ± 9	18 (7.5%)	88 (37.7%)	NA	9 (3.8%)	NA	
Mugnai et al. (16)	Belgium	Retrospective study	PAF or PerAF	473 (78.8%)	CB2/CB3	600 (500/100)	384 (64%)	58.1 ± 12.9	26.7 ± 4.6	58.1 ± 6.7	42.9 ± 9.3	51 (8%)	261 (43%)	53 (9%)	NA	12.6 ± 4.4 and 4.2 ± 1.4	6
Pott et al. (17)	Germany	Retrospective study	PAF or PerAF	40 (54.1%)	CB2/CB3	74 (37/37)	47 (64%)	65.3 ± 9.5	NA	NA	45 ± 7.9	12 (16.2%)	54 (73%)	22 (29.7%)	NA	6.2 ± 2.9	6
Sciarra et al. (18)	Indian	Cohort study	PAF or PerAF	46 (67.6%)	CB2/CB3	68 (34/34)	48 (70.6%)	57.8 ± 9.6	NA	57.9 ± 4.9	42.2 ± 4.9	1 (1.5%)	18 (26.5%)	NA	NA	12	7
Heeger et al. (12)	Germany	Retrospective study	PAF or PerAF	135 (45%)	CB2/CB4	300 (150/150)	182 (60.7%)	66.5 ± 11	NA	NA	NA	40 (13.3%)	204 (68%)	71 (23.7%)	NA	NA	5
Miyazaki et al. (21)	Japan	Retrospective study	PAF or PerAF	66 (73.3%)	CB2/CB4	90 (41/49)	58 (64.4%)	66.8 ± 11.1	NA	62.3 ± 10.5	38.8 ± 5.2	NA	NA	NA	NA	NA	6
Moltrasio et al. (20)	Italy	Retrospective study	PAF or PerAF	80 (80%)	CB2/CB4	100 (50/50)	72 (72.0%)	59.7 ± 12.1	26.2 ± 5.7	60.5 ± 8.4	NA	7 (7%)	58 (58%)	9 (9%)	6 (6%)	NA	7
Rottner et al. (13)	Germany	Retrospective study	PAF or PerAF	134 (67%)	CB2/CB4	200 (100/100)	127 (63.5%)	55.5 ± 3.9	NA	58.5 ± 4.5	44.1 ± 5.5	21 (10.5%)	114 (57%)	NA	8 (4%)	NA	6
Manfrin et al. (19)	Italy	Retrospective study	PAF or PerAF	274 (55.7%)	CB2/CB4	492 (246/246)	144 (29.3%)	61.4 ± 9.4	27.2 ± 4.4	58.4 ± 6.3	42 ± 9	37 (7.6%)	277 (56.3%)	5 (1.1%)	21 (4.4%)	12	7

AF, atrial fibrillation; PAF, paroxysmal atrial fibrillation; PerAF, persistent atrial fibrillation; CB2, second-generation cryoballoon; CB3, third-generation cryoballoon; CB4, fourth-generation cryoballoon; y, years; BMI, body mass index; LVEF, left ventricular ejection fraction; LAD, left atrium diameter; CAD, coronary artery disease; TIA, transient ischemic attack; NA, not available.

### 3.2 Meta-analysis

#### 3.2.1 Meta-analysis of the outcomes of procedure time, fluoroscopy time, and the success rate of PVI

**Figure 2:**
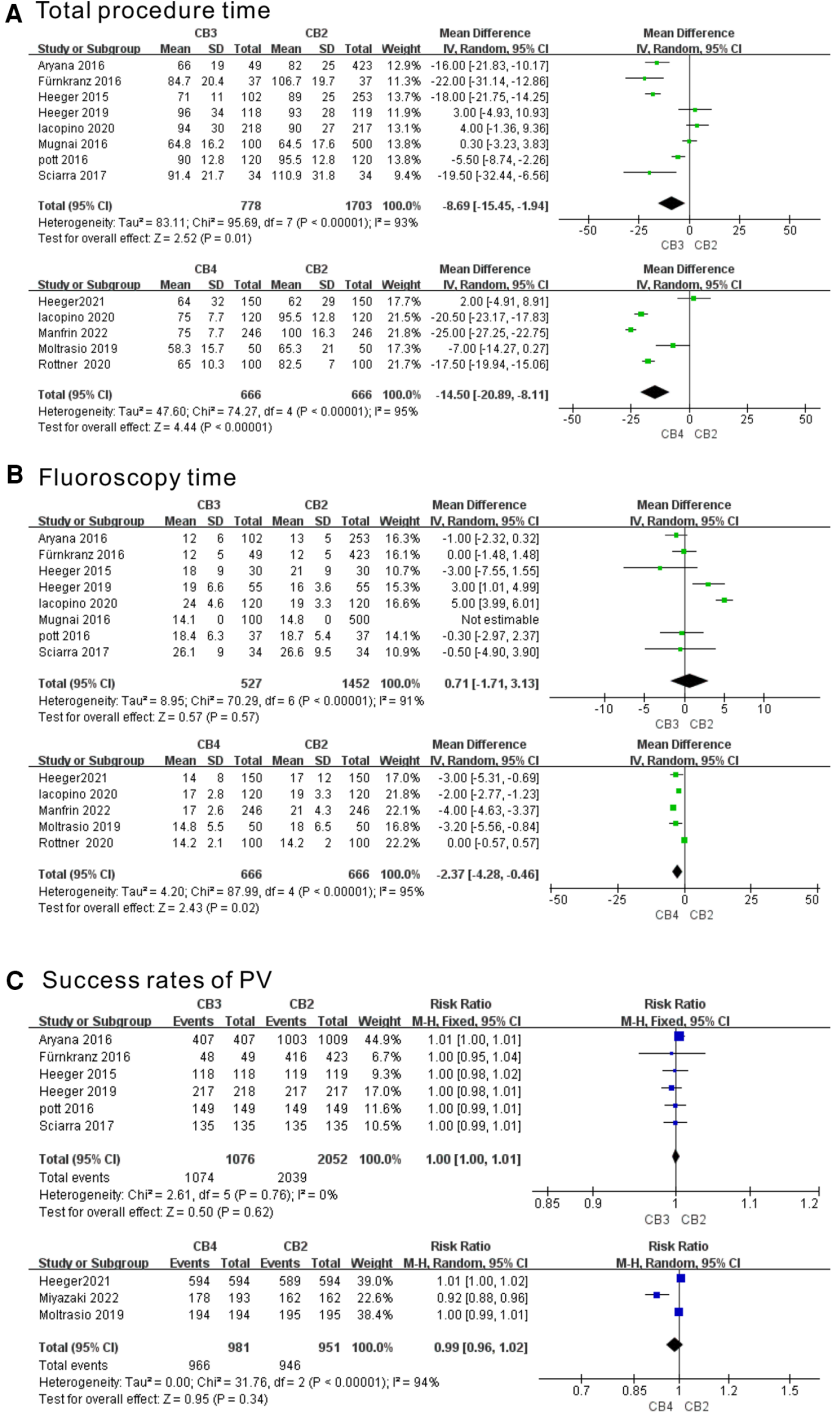
Forest plot of the outcome of total procedure time, fluoroscopy time, and success rate of PVI. (**A**) Total procedure time (min). (**B**) Fluoroscopy time (min). (**C**) Success rate of PVI. CB2, second-generation cryoballoon; CB3, third-generation cryoballoon; CB4, fourth-generation cryoballoon.

Eight and five studies evaluated the total procedure time of the CB3 and CB4 in the treatment of AF, respectively. As shown in Figure 2A, the CB3 had a significantly shorter procedure time than the CB2 (WMD = −8.69 min, 95% CI = −15.45 to −1.94 min, *P* < 0.01). As significant heterogeneity was observed (I^2^ = 93%, *p* < 0.00001), a random-effects model was applied. The CB4 had a significantly shorter procedure time than the CB2 (WMD = 14.50 min, 95% CI = −20.89 to −8.11 min, *P* < 0.00001). Eight and five studies evaluated the fluoroscopy time of the CB3 and CB4 in the treatment of AF, respectively. In terms of fluoroscopy time (Figure 2B), there was no significant difference between the CB3 and CB2 (WMD = 0.71 min, 95% CI = −1.71 to 3.13 min, *p* = 0.179), and the CB4 had a significantly shorter fluoroscopy time than the CB2 (WMD = −2.37 min, 95% CI = −4.28 to −0.46 min, *P* < 0.02). Five and three studies evaluated the success rate of PVI with the CB3 and CB4 in the treatment of AF, respectively. The success rates of PVI with CB3 vs. CB2 and CB4 vs. CB2 were as follows: RR = 1.00, 95% CI = 1.00–1.01 (*p* = 0.31), and RR = 0.99, 95% CI= 0.96–1.02 (*p* = 0.34), suggesting that neither the CB3 nor CB4 improved the success rate of PVI (Figure 2C). Although the PVI success rate of the CB3 was not heterogeneous (I^2 ^= 0%, *P* = 0.76), other studies showed great heterogeneity. The sensitivity analysis showed that there was no study that greatly affected the result of the meta-analysis (Supplementary Table S3).

#### 3.2.2 Meta-analysis of the outcome of the real-time PVI recordings

**Figure 3:**
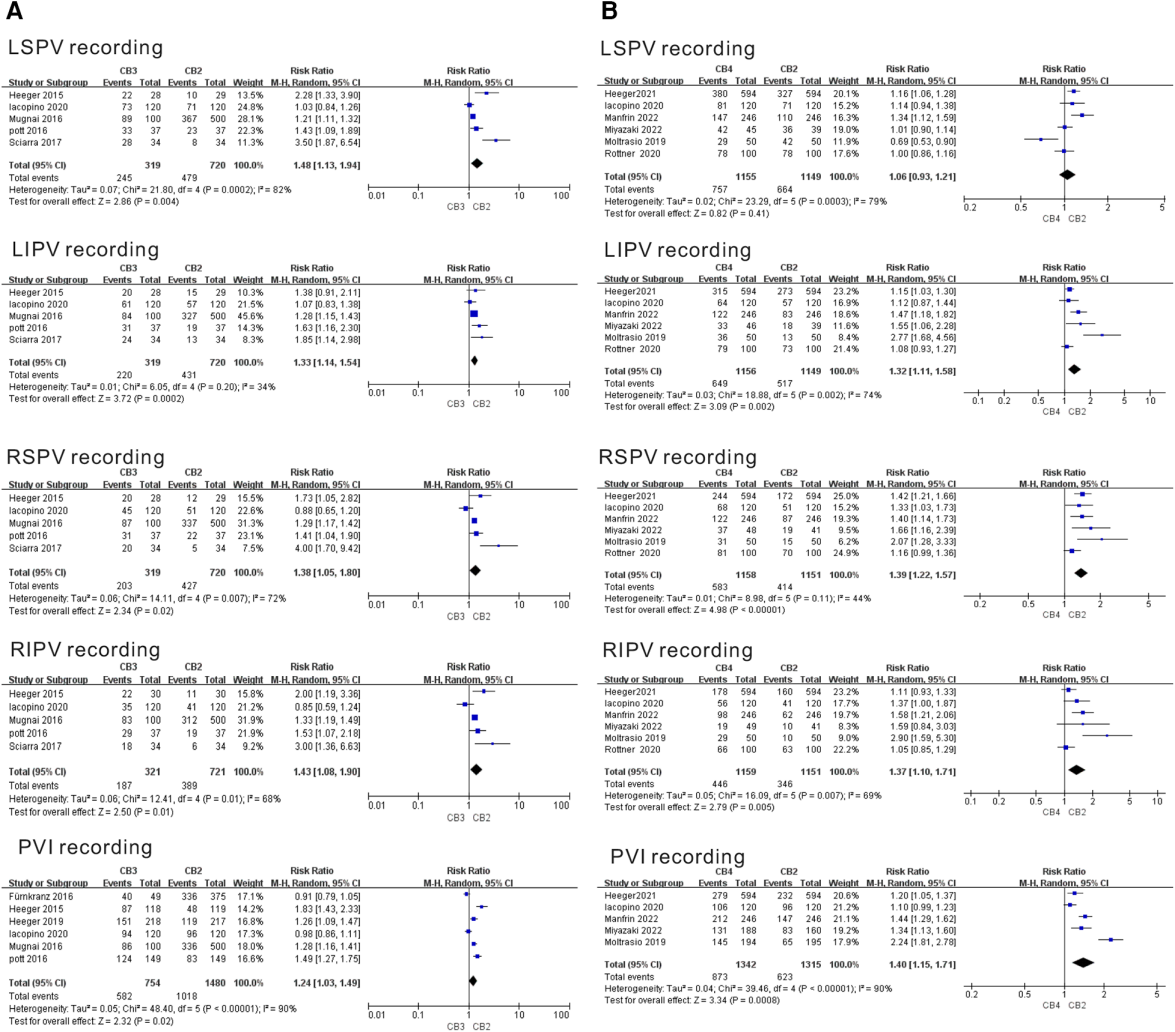
Forest plot of the outcome of real-time PVI recordings. (**A**) Real-time PVI recordings of CB3 vs. CB2. (**B**) Real-time PVI recordings of CB4 vs. CB2. CB2, second-generation cryoballoon; CB3, third-generation cryoballoon; CB4, fourth-generation cryoballoon.

Six separate studies evaluated the PVI recordings of the CB3 and CB4 in the treatment of AF. As shown in Figure 3A, during left superior pulmonary vein (LSPV) ablation, only the use of the CB3 increased the PVI recording compared with the CB2 group (CB3, RR = 1.48, 95% CI = 1.13–1.94, *P* = 0.004). During left inferior pulmonary vein (LIPV) ablation, the use of the CB3 and CB4 increased the PVI recording compared with the CB2 group (CB3, RR = 1.33, 95% CI = 1.14–1.54, *P* < 0.001; CB4, RR = 1.32, 95% CI = 1.11–1.58, *P* = 0.002). During right superior pulmonary vein (RSPV) ablation, the use of the CB3 and CB4 increased the PVI recording compared with the CB2 group (CB3, RR = 1.38, 95% CI = 1.05–1.80, *P* = 0.007; CB4, RR = 1.39, 95% CI = 1.27–1.57, *P* = 0.002). During right inferior pulmonary vein (RIPV) ablation, the use of the CB3 and CB4 increased the PVI recording compared with the CB2 group (CB3, RR = 1.43, 95% CI = 1.08–1.90, *P* = 0.01; CB4, RR = 1.37, 95% CI = 1.10–1.71, *P* < 0.001). During all PV ablations, the use of the CB3 and CB4 still increased the PVI recording compared with the CB2 group (CB3, RR = 1.24, 95% CI = 1.03–1.49, *P* < 0.001; CB4, RR = 1.40, 95% CI = 1.15–1.71, *P* < 0.03). Except for the study of moderate heterogeneity in the LIPV recording of the CB3 (I^2 ^= 34%, *P* = 0.02) and RSPV recording of the CB4 (I^2 ^= 44%, *P* < 0.001), other studies showed moderate heterogeneity. The sensitivity analysis showed that LIPV recording of heterogeneity in the CB4 group was significantly reduced after deleting the Moltrasio et al. (20) study (I^2 ^= 48%, *P* = 0.11). In addition, RSPV recording of heterogeneity in the CB4 group was reduced after deleting the Rottner et al. (13) (I^2 ^= 30%, *P* = 0.22) or Manfrin et al. (19) (I^2 ^= 0%, *P* = 0.52) studies (Supplementary Table S3). This indicated that heterogeneity might be derived from the above studies.

#### 3.2.3 Meta-analysis of the outcome of the TTI

**Figure 4:**
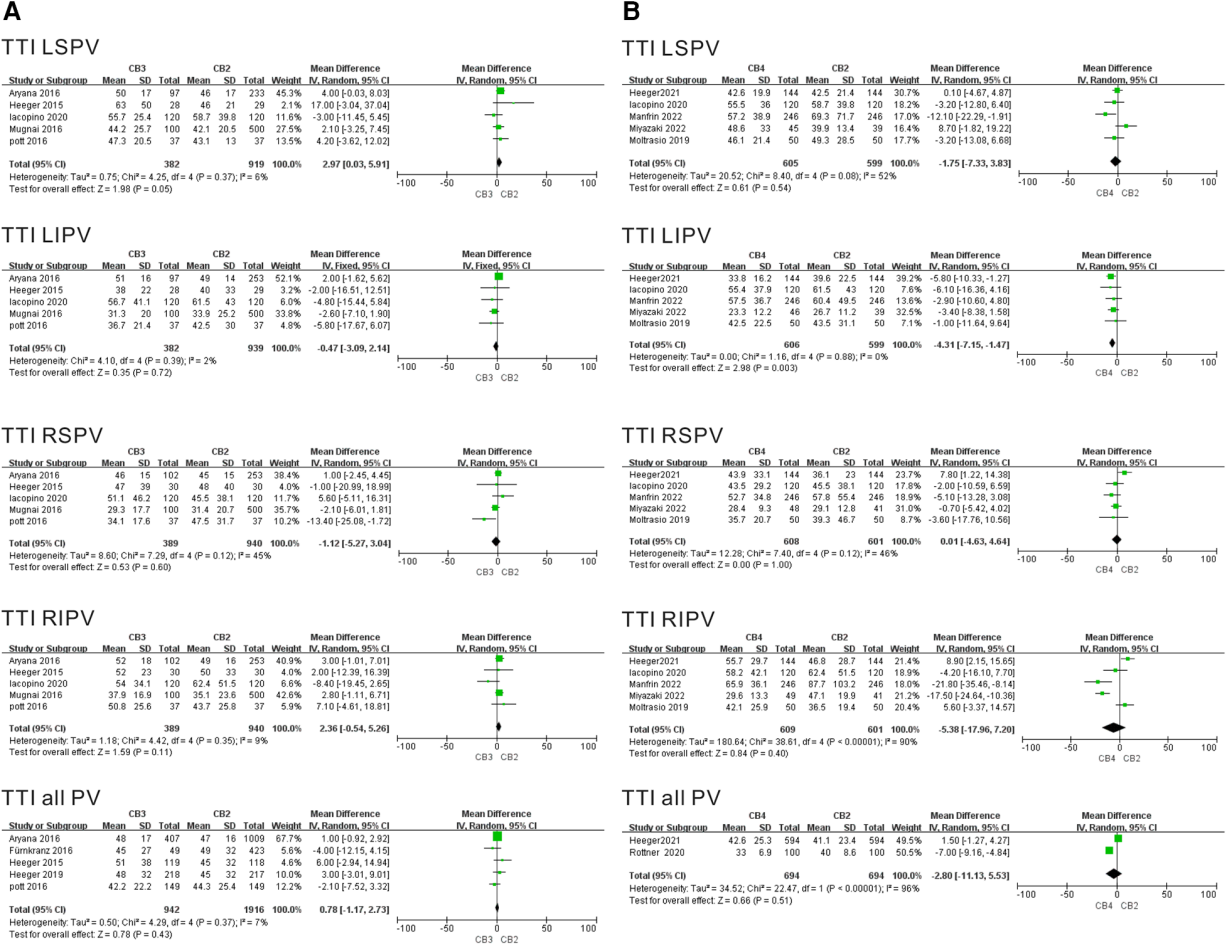
Forest plot of the outcome of TTI. (**A**) TTI of CB3 vs. CB2 (min). (**B**) TTI of CB4 vs. CB2 (min). TTI, time-to-isolation; CB2, second-generation cryoballoon; CB3, third-generation cryoballoon; CB4, fourth-generation cryoballoon.

Five and six separate studies evaluated the TTI of the CB3 and CB4 in the treatment of AF. The TTI results per individual PV ablation data per individual PV is summarized in Figure 4. The results showed that in the CB3 group, the TTI LSPV was greater than that in the CB2 group (WMD = 2.97 min, 95% CI = 0.03–5.91 min, *P* = 0.05), and there was lower heterogeneity in the results (I^2^ = 6%, *P* = 0.37). The results showed that in the CB4 group, the TTI LIPV was shorter than that in the CB2 group (WMD = −4.31 min, 95% CI = −7.15 to −1.47 min, *P* = 0.003), and there was no heterogeneity in the results (I^2 ^= 0%, *P* = 0.003). Conversely, there were no discernible differences in the duration of the TTI LIPV, TTI RSPV, or TTI RIPV between the CB3 and CB2. Moreover, TTI was also similar between the CB3 and CB2. The sensitivity analysis demonstrated that the TTI RIPV in studies of the CB3 vs. CB2 group showed a positive result after eliminating Iacopino et al. (WMD = 3.09 min, 95% CI = 0.41–5.76 min, *P* = 0.02) (11). The results of TTI LIPV in the CB4 vs. CB2 study was positive after excluding Iacopino et al. (11) (WMD = −4.16 min, 95% CI = −7.11 to −1.21 min, *P* = 0.006), Manfrin et al. (19) (WMD = −4.53 min, 95% CI = −7.58 to −1.48 min, *P* = 0.004), Miyazaki et al. (21) (WMD = −4.75 min, 95% CI = −8.20 to −1.30 min, *P* = 0.007), and Moltrasio et al. (20) (WMD = −4.56 min, 95% CI = −7.51 to −1.62 min, *P* = 0.02) in turn (Supplementary Table S3).

#### 3.2.4 Meta-analysis of the outcome of the mean nadir temperature

**Figure 5:**
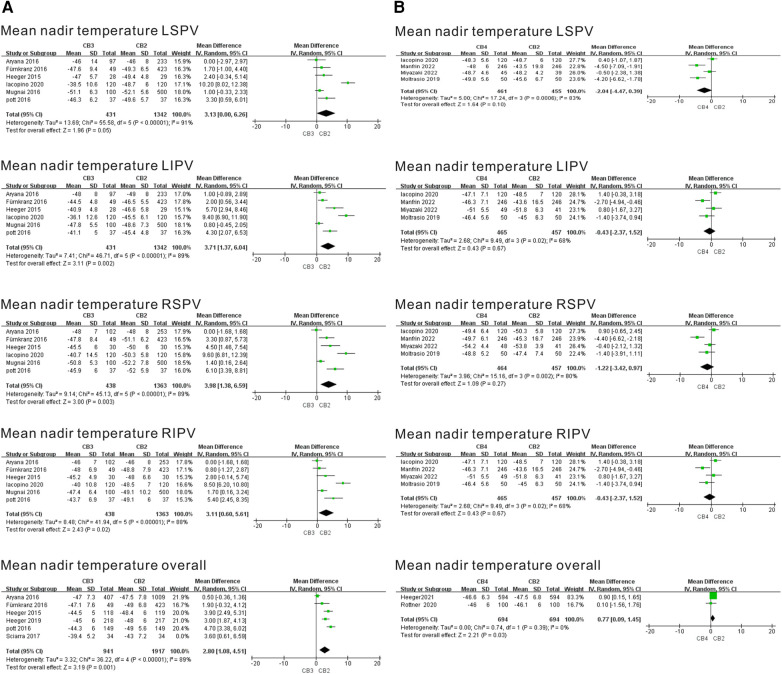
Forest plot of the outcome of mean nadir temperature. (**A**) Mean nadir temperature of CB3 vs. CB2 (°C). (**B**) Mean nadir temperature of CB4 vs. CB2 (°C). TTI, time-to-isolation; CB2, second-generation cryoballoon; CB3, third-generation cryoballoon; CB4, fourth-generation cryoballoon.

Six and five separate studies evaluated the mean nadir temperature of the CB3 and CB4 in the treatment of AF. Mean nadir temperature results per individual PV ablation data per individual PV are summarized in Figure 5. As shown in the CB3 vs. CB2 study, the mean nadir temperature was significantly more prevalent in the CB3 group compared with the CB2 group. Applying a TTI-guided ablation strategy, the mean nadir temperature was noted for the CB3 targeting the LSPV (WMD = 3.13℃, 95% CI = 0.00℃–6.26℃, *P* = 0.05), the CB3 targeting the LIPV (WMD = 3.71℃, 95% CI = 1.37℃–6.04℃, *p* = 0.002), the CB3 targeting the RSPV (WMD = 3.98℃, 95% CI = 1.38℃–6.59℃, *P* = 0.003), the CB3 targeting the RIPV (WMD = 3.11℃, 95% CI = 0.60℃–5.61℃, *P* = 0.02), and the CB3 targeting the overall PVs (WMD = 2.80℃, 95% CI = 1.08℃–4.51℃, *P* = 0.001). As shown in the CB4 vs. CB2 study, the mean nadir temperature was not significantly more prevalent in the CB4 group compared with the CB2 group, and no difference was found for the other PVs. The sensitivity analysis showed that the overall mean nadir temperature in studies of the CB3 vs. CB2 group was significantly reduced after eliminating the Aryana et al. (9) study (I^2^ = 35%, *P* = 0.19) (Supplementary Table S3). This indicated that heterogeneity might be derived from this study.

#### 3.2.5 Meta-analysis of the outcome of the AF recrudescence and complications

**Figure 6:**
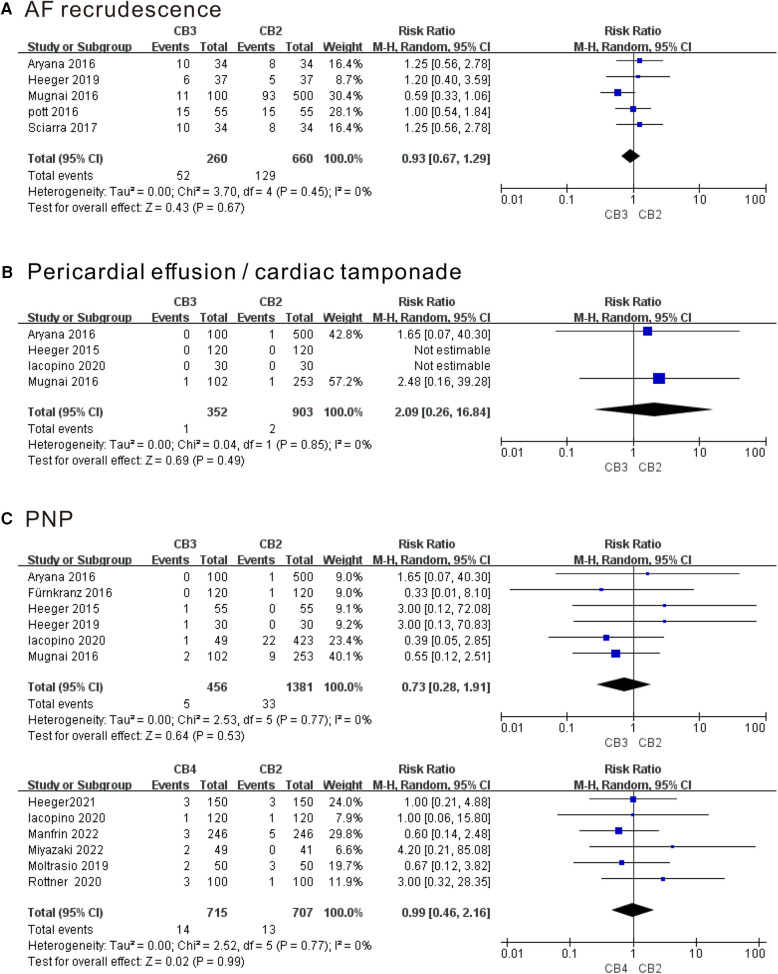
Forest plot of the outcome of AF recrudescence and complications. (**A**) AF recurrence. (**B**) Pericardial effusion/cardiac tamponade. (**C**) PNP. AF, atrial fibrillation; PNP, phrenic nerve palsy; CB2, second-generation cryoballoon; CB3, third-generation cryoballoon; CB4, fourth-generation cryoballoon.

As shown in Figure 6, compared with the CB2 group, PVI using CB3 or CB4 did not show fewer AF recurrences, pericardial effusion/cardiac tamponade as well as PNP at follow-up time (*P* > 0.05). There was no heterogeneity among the studies mentioned above. In addition, the rate of other serious adverse events in our series was considerably low. Groin complications only occurred in two patients in the CB3 group, one patient in the CB4 group, and eight patients in the CB2 group. One patient in each CB2 group developed atrioesophageal fistula and stroke/transient ischemic attack. No symptomatic PV stenosis occurred. Sensitivity analysis showed that no single study had a significant impact on the results of the meta-analysis (Supplementary Table S3).

#### 3.2.6 Analysis of publication bias

The risk of publication bias was evaluated using a funnel plot (Supplementary Figures S1 and S2) with regard to the outcomes of TTI LIPV, mean nadir temperature LSPV, and overall mean nadir temperature in CB3 research. No evidence of potential publication bias was revealed for outcomes in CB3 and CB4 research.

## 4 Discussion

This meta-analysis investigated the safety and efficacy of the CB3 or CB4 compared with the CB2 for PVI in AF patients. The main findings were as follows: (1) the CB3 and CB4 were safe and efficacious in PVI for the treatment of AF; (2) CB3 or CB4 ablation did not reduce the risk of AF recrudescence and complications, including PNP and pericardial effusion/pericardial tamponade; (3) and the use of the CB3 seemed to reduce the total procedure time, increase the PVI recording, and increase the mean minimum temperature, and the use of the CB4 reduced the total procedure time and fluoroscopy time, and increased PVI recording. In this regard, the findings of the present meta-analysis provided relatively more comprehensive evidence that might be used to inform further clinical decision-making in the treatment of AF ablation.

This study showed that the total procedure times of the CB3 and CB4 groups were shorter than those of the CB2 group. The possible explanation for this is that the advanced design of the CB3 and CB4 groups improved contact with the PV sinus, resulting in faster PVI. Our study found that real-time recording of PV isolation was possible at much higher rates with the CB3 or CB4 compared with the CB2 group. A high live PV signal rate during ablation led to better outcomes, which prevented edema formation, avoided overtreatment when electrical isolation was achieved, and reduced the risk of imperfect lesion formation caused by an ineffective freeze cycle (22). TTI was an important predictor of AF recurrence, and rapid TTI within 60 s was also confirmed to indicate long-lasting isolation (23). However, the sensitivity analysis showed that the results of TTI RIPV in the CB3 vs. CB2 group and TTI LIPV in the CB4 vs. CB2 group were unstable after excluding some studies. Therefore, the TTI of the CB3 or CB4 still needs to be further elucidated. However, excessive ablation may lead to the formation of lesions in the lungs or esophagus. The visibility of potential recordings from the PV is crucial in this context (24). The distal tips of the CB3 and CB4 were 40% shorter than that of the CB2 (14 vs. 8 mm, respectively), which has greatly shortened the distance between the ablation site and the inner lumen mapping catheter position (25, 26). Therefore, it promoted the operability of the catheter and increased the possibility of venous signal recording during ablation. This meta-analysis demonstrated that CB3 exhibited higher temperatures and offered improved safety during ablation. The balloon temperature played a pivotal role in predicting PV reconnection during the index procedure and AF recurrence following CBA (27). In cases in which TTI cannot be recorded, temperature can be used as a reliable measure to evaluate the effectiveness of PVI. However, it is important to consider the impact of temperature on the safety of cryoablation. If the freezing temperature was too low, it might result in damage to the extrapulmonary vein tissue and increase the risk of complications. Hence, the surgical operator would strengthen the standardized training to improve the safety and success rate of the AF ablation. We also noted a new cryoballoon, the POLARx (Boston Scientific, St. Paul, MN, USA), which was technically modified to improve patient outcomes. The multiple previous studies (28–30) proved that the novel POLARx cryoablation showed similar safety and efficacy to the CB4, even showing a higher rate of real-time PV recordings and lower balloon temperature in the Heeger et al. (28) study. Moreover, the balloon diameter of the POLARx FIT system would be expanded from 28 to 31 mm, which improved the ablation area including the pulmonary vein protuberance, and is comparable with the existing POLARx system for AF treatment (31).

With the CBA procedure, freedom from recrudescence was often the most important measurement of its efficacy. AF, atrial flutter, or atrial tachycardia that occurred 3 months after ablation and lasted longer than 30 s was considered as AF recurrence. A previous study showed that anti-arrhythmic drug therapy decreased from 71.7% before CB2 ablation to 33.6% post-ablation, and the freedom from symptomatic AF recurrence was 78% for paroxysmal AF and 77% for persistent AF (32). Our study proved that a follow-up time recrudescence rate of PVI using the CB3 was not higher than the success rates of CB2. The AF recurrence data of the CB4 were insufficient to conduct a meta-analysis but a previous study of the CB4 demonstrated a significant reduction in the recurrence rate of AF among patients treated with CB4 PVI during the 12-month follow-up (19). Our study indicated that the complication rate of the CB3 or CB4 was very low, demonstrating the safety of the procedure, especially for pericardial effusion/cardiac tamponade, PNP, groin complications, atrioesophageal fistula, stroke/transient ischemic attack, and symptomatic PV stenosis. Improper operation of the cryoballoon in the left atrium might lead to left atrial perforation or laceration leading to pericardial tamponade, but the probability of cardiac tamponade in CBA was low. Only one patient and two patients in the CB4 and CB2 had pericardial effusion or pericardial tamponade, respectively (19). Pericardial effusion/cardiac tamponade is a common complication associated with this procedure. Asymptomatic pericardial effusion/cardiac tamponade can be identified through a basic echocardiographical examination. Fortunately, it was demonstrated that pericardial effusion/cardiac tamponade after AF ablation was mainly mild, and most clinical outcomes were benign (33). In our population, approximately 1.1% and 2.0% of patients developed PNP after receiving CB3 and CB4 ablation, respectively. PNP was a common complication that could occur during PVI using the cryoballoon and had the potential to negate the clinical benefits of a restored sinus rhythm. Mild PNP is asymptomatic; patients with severe PNP may present with clinical signs such as dyspnea and shortness of breath with activity. Although most patients would recover during postoperative follow-up, the prevention of PNP was still the focus of CBA in the treatment of AF. The distance between the ablation site and the phrenic nerve was also an important factor in PNP occurrence during catheter ablation (34). Female gender and a lower BMI might be independent predictors of non-transient PNP, and hence diaphragmatic compound motor action potentials should be monitored in this patient population (35, 36). Furthermore, previous studies reported that the application of the intracardiac echocardiography technique reduces the risk of freezing greatly in the vein and the risk of PNP by providing confirmation of the correct positioning of the balloon (37).

It should be mentioned that a certain degree of heterogeneity between the pooled studies was observed in the present study. Several factors may have contributed to this heterogeneity. First, all the data used to evaluate the safety and efficacy of CBA were derived from retrospective rather than randomized studies. The final outcomes were vulnerable to unmeasured unnoticed biases and confounding, even after complex statistical adjustments. Therefore, large-scale double-blind randomization trials are greatly warranted to further verify our results and confirm the safety and efficacy of CBA. Second, safety and efficacy were less exactly defined due to the BMI, LVEF, follow-up time, and history-taking paucity of some data in the literature. The follow-up time in the original studies was also different. Presently, early recurrences of atrial tachyarrhythmias within a blanking period would not be considered when evaluating AF recurrence (38), given the fact that inflammation was one of the reasons for the early recurrence of AF after CBA. Moreover, this meta-analysis did not advance to PROSPERO registration. Finally, the sample size was still relatively small and may not be powered to precisely estimate the clinical outcomes. More studies with larger sample sizes are hence suggested to offer a more representative analysis. These limitations should be noted and addressed in future clinical investigations.

## 5 Conclusions

Our meta-analysis demonstrated a greater improvement in total procedure time, PVI recording, mean nadir temperature for AF patients referred for the CB3, and total procedure time, fluoroscopy time, and PVI recording for the CB4 compared with the CB2. However, it should be noted that the TTI, success rate of PVI, AF recurrence, and complications in the CB3 and CB4 were comparable with the CB2. In this regard, we recommend that large prospective randomized controlled studies should be performed in the future to validate our results.

## Data Availability

The raw data supporting the conclusions of this article will be made available by the authors, without undue reservation.
