# Supplementary material for: The safety and efficacy of third- and fourth-generation cryoballoons for atrial fibrillation: a systematic review and meta-analysis
Source: Front Cardiovasc Med. 2024 Aug 12;11:1364893. doi: 10.3389/fcvm.2024.1364893 (PMC11345166; doi:10.3389/fcvm.2024.1364893)
Supplement: Supplementary file 1 [file Datasheet1.docx]

Supplementary Material


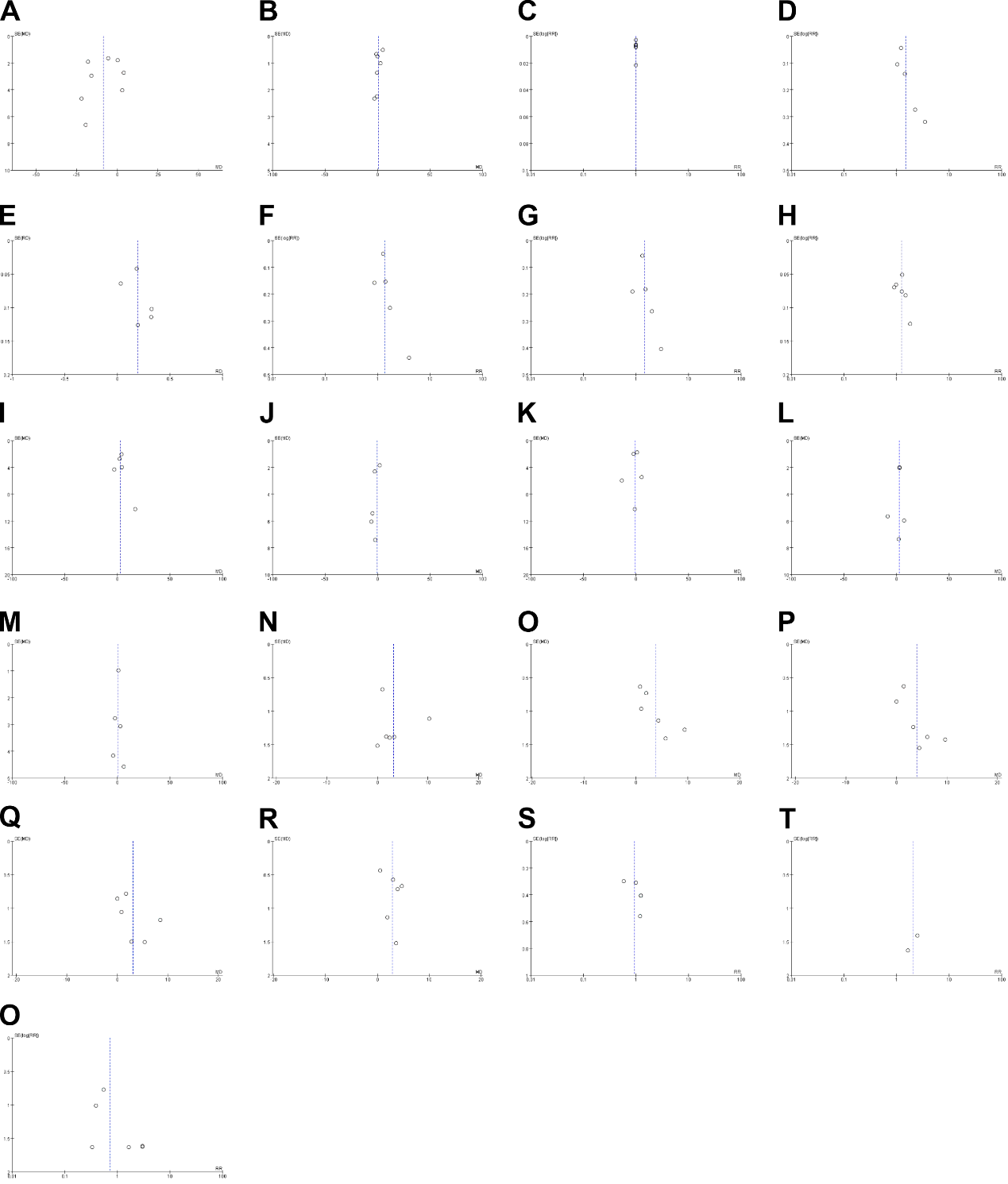


Figure S1. Funnel plot of all studies on CB3 vs CB2. (A) Total procedure time; (B) fluoroscopy time; (C) success rate of PVI; (D) LSPV recording; (E) LIPV recording; (F) RSPV recording; (G) LIPV recording; (H) PVI recording; (I) TTI LSPV; (J) TTI LIPV; (K) TTI RSPV; (L) TTI RIPV; (M) TTI all PV; (N) mean nadir temperature LSPV; (O) mean nadir temperature LIPV; (P) mean nadir temperature RSPV; (Q) mean nadir temperature RIPV; (R) mean nadir temperature overall; (S) AF recurrence; (T) pericardial effusion / cardiac tamponade; (O) PNP.

**
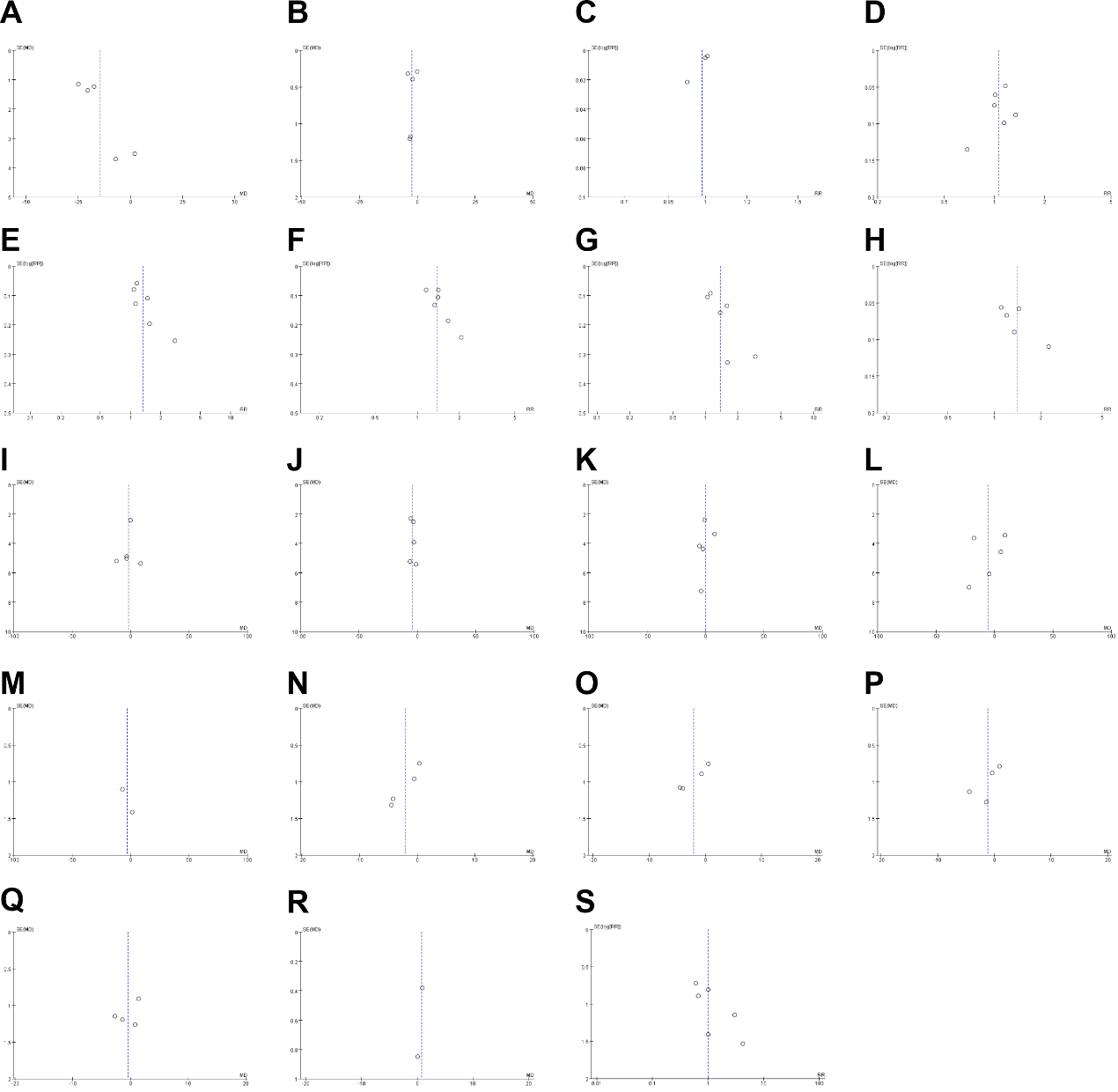
**

Figure S2. Funnel plot of all studies on CB4 vs CB2. (A) Total procedure time; (B) fluoroscopy time; (C) success rate of PVI; (D) LSPV recording; (E) LIPV recording; (F) RSPV recording; (G) LIPV recording; (H) PVI recording; (I) TTI LSPV; (J) TTI LIPV; (K) TTI RSPV; (L) TTI RIPV; (M) TTI all PV; (N) mean nadir temperature LSPV; (O) mean nadir temperature LIPV; (P) mean nadir temperature RSPV; (Q) mean nadir temperature RIPV; (R) mean nadir temperature overall; (S) PNP.

Table S1. PRISMA 2020 checklist.

| **Section and Topic** | **Item #** | **Checklist item** | **Location where item is reported** |
| --- | --- | --- | --- |
| **TITLE** | | |  |
| Title | 1 | Identify the report as a systematic review. | lines 60-69 |
| **ABSTRACT** | | |  |
| Abstract | 2 | See the PRISMA 2020 for Abstracts checklist. | lines 79-105 |
| **INTRODUCTION** | | |  |
| Rationale | 3 | Describe the rationale for the review in the context of existing knowledge. | lines 115-168 |
| Objectives | 4 | Provide an explicit statement of the objective(s) or question(s) the review addresses. | lines 169-173 |
| **METHODS** | | |  |
| Eligibility criteria | 5 | Specify the inclusion and exclusion criteria for the review and how studies were grouped for the syntheses. | lines 198-217 |
| Information sources | 6 | Specify all databases, registers, websites, organisations, reference lists and other sources searched or consulted to identify studies. Specify the date when each source was last searched or consulted. | lines 180-188 |
| Search strategy | 7 | Present the full search strategies for all databases, registers and websites, including any filters and limits used. | lines 178-195 |
| Selection process | 8 | Specify the methods used to decide whether a study met the inclusion criteria of the review, including how many reviewers screened each record and each report retrieved, whether they worked independently, and if applicable, details of automation tools used in the process. | lines 178-195 |
| Data collection process | 9 | Specify the methods used to collect data from reports, including how many reviewers collected data from each report, whether they worked independently, any processes for obtaining or confirming data from study investigators, and if applicable, details of automation tools used in the process. | lines 188-195 |
| Data items | 10a | List and define all outcomes for which data were sought. Specify whether all results that were compatible with each outcome domain in each study were sought (e.g. for all measures, time points, analyses), and if not, the methods used to decide which results to collect. | lines 225-233 |
|  | 10b | List and define all other variables for which data were sought (e.g. participant and intervention characteristics, funding sources). Describe any assumptions made about any missing or unclear information. | lines 233-236 |
| Study risk of bias assessment | 11 | Specify the methods used to assess risk of bias in the included studies, including details of the tool(s) used, how many reviewers assessed each study and whether they worked independently, and if applicable, details of automation tools used in the process. | lines 269-270 |
| Effect measures | 12 | Specify for each outcome the effect measure(s) (e.g. risk ratio, mean difference) used in the synthesis or presentation of results. | lines 253-256 |
| Synthesis methods | 13a | Describe the processes used to decide which studies were eligible for each synthesis (e.g. tabulating the study intervention characteristics and comparing against the planned groups for each synthesis (item #5)). | lines 236-239 |
|  | 13b | Describe any methods required to prepare the data for presentation or synthesis, such as handling of missing summary statistics, or data conversions. | lines 233-236 |
|  | 13c | Describe any methods used to tabulate or visually display results of individual studies and syntheses. | lines 253-256 |
|  | 13d | Describe any methods used to synthesize results and provide a rationale for the choice(s). If meta-analysis was performed, describe the model(s), method(s) to identify the presence and extent of statistical heterogeneity, and software package(s) used. | lines 258-265 |
|  | 13e | Describe any methods used to explore possible causes of heterogeneity among study results (e.g. subgroup analysis, meta-regression). | lines 265-270 |
|  | 13f | Describe any sensitivity analyses conducted to assess robustness of the synthesized results. | lines 265-269 |
| Reporting bias assessment | 14 | Describe any methods used to assess risk of bias due to missing results in a synthesis (arising from reporting biases). | lines 269-170 |
| Certainty assessment | 15 | Describe any methods used to assess certainty (or confidence) in the body of evidence for an outcome. | lines 252-256 |
| **RESULTS** | | |  |
| Study selection | 16a | Describe the results of the search and selection process, from the number of records identified in the search to the number of studies included in the review, ideally using a flow diagram. | Fig 1 |
|  | 16b | Cite studies that might appear to meet the inclusion criteria, but which were excluded, and explain why they were excluded. | Uninvolved |
| Study characteristics | 17 | Cite each included study and present its characteristics. | Table 1 |
| Risk of bias in studies | 18 | Present assessments of risk of bias for each included study. | Fig S1-S2 |
| Results of individual studies | 19 | For all outcomes, present, for each study: (a) summary statistics for each group (where appropriate) and (b) an effect estimate and its precision (e.g. confidence/credible interval), ideally using structured tables or plots. | Fig 1-6, Table S3 |
| Results of syntheses | 20a | For each synthesis, briefly summarise the characteristics and risk of bias among contributing studies. | lines 175-294 and lines 838-878 |
|  | 20b | Present results of all statistical syntheses conducted. If meta-analysis was done, present for each the summary estimate and its precision (e.g. confidence/credible interval) and measures of statistical heterogeneity. If comparing groups, describe the direction of the effect. | lines 299-836 |
|  | 20c | Present results of all investigations of possible causes of heterogeneity among study results. | lines 387-429 and lines 781-821 |
|  | 20d | Present results of all sensitivity analyses conducted to assess the robustness of the synthesized results. | Table S3 |
| Reporting biases | 21 | Present assessments of risk of bias due to missing results (arising from reporting biases) for each synthesis assessed. | lines 838-878 |
| Certainty of evidence | 22 | Present assessments of certainty (or confidence) in the body of evidence for each outcome assessed. | lines 291-294 |
| **DISCUSSION** | | |  |
| Discussion | 23a | Provide a general interpretation of the results in the context of other evidence. | lines 883-896 |
|  | 23b | Discuss any limitations of the evidence included in the review. | lines 1185-1207 |
|  | 23c | Discuss any limitations of the review processes used. | lines 1185-1207 |
|  | 23d | Discuss implications of the results for practice, policy, and future research. | lines 1212-1220 |
| **OTHER INFORMATION** | | |  |
| Registration and protocol | 24a | Provide registration information for the review, including register name and registration number, or state that the review was not registered. | The review was not registered. |
|  | 24b | Indicate where the review protocol can be accessed, or state that a protocol was not prepared. | The protocol was not prepared |
|  | 24c | Describe and explain any amendments to information provided at registration or in the protocol. | The review was not registered. |
| Support | 25 | Describe sources of financial or non-financial support for the review, and the role of the funders or sponsors in the review. | lines 1230-1239 |
| Competing interests | 26 | Declare any competing interests of review authors. | lines 1244-1247 |
| Availability of data, code and other materials | 27 | Report which of the following are publicly available and where they can be found: template data collection forms; data extracted from included studies; data used for all analyses; analytic code; any other materials used in the review. | lines 1224-1226 |

Table S2. Detailed search queries for each database.

| Number/database | Key words | Count |
| --- | --- | --- |
| Dentification |  |  |
| Pubmed | fourth generation cryoballoon and atrial fibrillation | 23 |
| Web of sci | fourth generation cryoballoon and atrial fibrillation | 21 |
| Cochrane | fourth generation cryoballoon and atrial fibrillation | 2 |
| CNKI/Wangfang/VIP | fourth generation cryoballoon and atrial fibrillation | 0 |
| Pubmed | third generation cryoballoon and atrial fibrillation | 36 |
| Web of sci | third generation cryoballoon and atrial fibrillation | 36 |
| Cochrane | third generation cryoballoon and atrial fibrillation | 11 |
| CNKI/Wangfang/VIP | third generation cryoballoon and atrial fibrillation | 0 |
| Total |  | 129 |
| Exclusion of duplicate studies |  | -58 |
| Articles that were not relevant to the purpose of this study were excluded |  | -30 |
| The articles without relevant data were excluded |  | -28 |
| Eligible articles |  | 13 |

Table S3. Sensitivity analysis of all outcomes.

| CB3 vs CB2 | | | | CB4 vs CB2 | | | |
| --- | --- | --- | --- | --- | --- | --- | --- |
| Excluded study | Proportion (95% CI) | Test for overall effect | Heterogeneity | Excluded study | Proportion (95% CI) | Test for overall effect | Heterogeneity |
| Total procedure time | | | | | | | |
| Aryana 2016 | -7.62 [-14.93, -0.31] | Z = 2.04 (P = 0.04) | Tau² = 84.92; Chi² = 85.88, df = 6 (P < 0.00001); I² = 93% | Heeger2021 | -18.47 [-23.52, -13.42] | Z = 7.17 (P < 0.00001) | Tau² = 22.75; Chi² = 34.27, df = 3 (P < 0.00001); I² = 91% |
| Fürnkranz 2016 | -6.98 [-13.95, 0.00] | Z = 1.96 (P = 0.05) | Tau² = 78.04; Chi² = 85.10, df = 6 (P < 0.00001); I² = 93% | Iacopino 2020 | -12.50 [-21.53, -3.48] | Z = 2.72 (P = 0.007) | Tau² = 77.88; Chi² = 73.98, df = 3 (P < 0.00001); I² = 96% |
| Heeger 2015 | -7.03 [-13.40, -0.66] | Z = 2.16 (P = 0.03) | Tau² = 61.03; Chi² = 54.69, df = 6 (P < 0.00001); I² = 89% | Manfrin 2022 | -11.54 [-18.94, -4.15] | Z = 3.06 (P = 0.002) | Tau² = 50.12; Chi² = 43.06, df = 3 (P < 0.00001); I² = 93% |
| Heeger 2019 | -10.28 [-17.49, -3.07] | Z = 2.79 (P = 0.005) | Tau² = 83.45; Chi² = 89.17, df = 6 (P < 0.00001); I² = 93% | Moltrasio 2019 | -16.15 [-22.82, -9.48] | Z = 4.75 (P < 0.00001) | Tau² = 42.48; Chi² = 61.79, df = 3 (P < 0.00001); I² = 95% |
| Iacopino 2020 | -10.58 [-17.62, -3.55] | Z = 2.95 (P = 0.003) | Tau² = 77.64; Chi² = 77.41, df = 6 (P < 0.00001); I² = 92% | Rottner 2020 | -13.29 [-22.34, -4.25] | Z = 2.88 (P = 0.004) | Tau² = 78.22; Chi² = 69.04, df = 3 (P < 0.00001); I² = 96% |
| Mugnai 2016 | -10.14 [-17.58, -2.70] | Z = 2.67 (P = 0.008) | Tau² = 87.46; Chi² = 73.73, df = 6 (P < 0.00001); I² = 92% |  |  |  |  |
| Pott 2016 | -9.33 [-17.87, -0.79] | Z = 2.14 (P = 0.03) | Tau² = 119.05; Chi² = 94.40, df = 6 (P < 0.00001); I² = 94% |  |  |  |  |
| Sciarra 2017 | -7.57 [-14.66, -0.49] | Z = 2.10 (P = 0.04) | Tau² = 82.78; Chi² = 92.09, df = 6 (P < 0.00001); I² = 93% |  |  |  |  |
| Fluoroscopy time | | | | | | | |
| Aryana 2016 | 1.06 [-1.50, 3.62] | Z = 0.81 (P = 0.42) | Tau² = 8.25; Chi² = 45.49, df = 5 (P < 0.00001); I² = 89% | Heeger2021 | -2.24 [-4.39, -0.10] | Z = 2.05 (P = 0.04) | Tau² = 4.41; Chi² = 87.11, df = 3 (P < 0.00001); I² = 97% |

**Table S2.** *Continued*

| CB3 vs CB2 | | | | CB4 vs CB2 | | | |
| --- | --- | --- | --- | --- | --- | --- | --- |
| Excluded study | Proportion (95% CI) | Test for overall effect | Heterogeneity | Excluded study | Proportion (95% CI) | Test for overall effect | Heterogeneity |
| Fluoroscopy time | | | | | | | |
| Fürnkranz 2016 | 0.80 [-2.07, 3.67] | Z = 0.55 (P = 0.58) | Tau² = 10.85; Chi² = 62.23, df = 5 (P < 0.00001); I² = 92% | Iacopino 2020 | -2.50 [-5.16, 0.16] | Z = 1.84 (P = 0.07) | Tau² = 6.66; Chi² = 87.92, df = 3 (P < 0.00001); I² = 97% |
| Heeger 2015 | 1.15 [-1.38, 3.69] | Z = 0.89 (P = 0.37) | Tau² = 8.73; Chi² = 65.69, df = 5 (P < 0.00001); I² = 92% | Manfrin 2022 | -1.80 [-3.39, -0.20] | Z = 2.20 (P = 0.03) | Tau² = 2.04; Chi² = 24.20, df = 3 (P < 0.0001); I² = 88% |
| Heeger 2019 | 0.26 [-2.57, 3.10] | Z = 0.18 (P = 0.86) | Tau² = 10.60; Chi² = 69.07, df = 5 (P < 0.00001); I² = 93% | Moltrasio 2019 | -2.20 [-4.34, -0.06] | Z = 2.02 (P = 0.04) | Tau² = 4.39; Chi² = 86.81, df = 3 (P < 0.00001); I² = 97% |
| Iacopino 2020 | 0.71 [-1.71, 3.13] | Z = 0.57 (P = 0.57) | Tau² = 8.95; Chi² = 70.29, df = 6 (P < 0.00001); I² = 91% | Rottner 2020 | -3.05 [-4.38, -1.71] | Z = 4.48 (P < 0.00001) | Tau² = 1.28; Chi² = 15.49, df = 3 (P = 0.001); I² = 81% |
| Mugnai 2016 | 0.86 [-1.83, 3.55] | Z = 0.63 (P = 0.53) | Tau² = 9.56; Chi² = 67.45, df = 5 (P < 0.00001); I² = 93% |  |  |  |  |
| Pott 2016 | 0.86 [-1.83, 3.55] | Z = 0.63 (P = 0.53) | Tau² = 9.56; Chi² = 67.45, df = 5 (P < 0.00001); I² = 93% |  |  |  |  |
| Sciarra 2017 | 0.85 [-1.75, 3.46] | Z = 0.64 (P = 0.52) | Tau² = 9.24; Chi² = 69.09, df = 5 (P < 0.00001); I² = 93% |  |  |  |  |
| Success rates of PV | | | | | | | |
| Aryana 2016 | 1.00 [0.99, 1.01] | Z = 0.46 (P = 0.65) | Chi² = 0.38, df = 4 (P = 0.98); I² = 0% | Heeger2021 | 0.96 [0.94, 0.98] | Z = 3.58 (P = 0.0003) | Chi² = 57.02, df = 1 (P < 0.00001); I² = 98% |
| Fürnkranz 2016 | 1.00 [1.00, 1.01] | Z = 0.71 (P = 0.48) | Chi² = 2.42, df = 4 (P = 0.66); I² = 0% | Miyazaki 2022 | 1.01 [1.00, 1.01] | Z = 1.89 (P = 0.06) | Chi² = 1.79, df = 1 (P = 0.18); I² = 44% |

**Table S2.** *Continued*

| CB3 vs CB2 | | | | CB4 vs CB2 | | | |
| --- | --- | --- | --- | --- | --- | --- | --- |
| Excluded study | Proportion (95% CI) | Test for overall effect | Heterogeneity | Excluded study | Proportion (95% CI) | Test for overall effect | Heterogeneity |
| Success rates of PV | | | | | | | |
| Heeger 2015 | 1.00 [1.00, 1.01] | Z = 0.52 (P = 0.60) | Chi² = 2.53, df = 4 (P = 0.64); I² = 0% | Moltrasio 2019 | 0.99 [0.98, 1.00] | Z = 1.95 (P = 0.05) | Chi² = 32.99, df = 1 (P < 0.00001); I² = 97% |
| Heeger 2019 | 1.00 [1.00, 1.01] | Z = 0.87 (P = 0.39) | Chi² = 1.22, df = 4 (P = 0.87); I² = 0% |  |  |  |  |
| Pott 2016 | 1.00 [1.00, 1.01] | Z = 0.52 (P = 0.60) | Chi² = 2.50, df = 4 (P = 0.65); I² = 0% |  |  |  |  |
| Sciarra 2017 | 1.00 [1.00, 1.01] | Z = 0.52 (P = 0.60) | Chi² = 2.51, df = 4 (P = 0.64); I² = 0% |  |  |  |  |
| LSPV recording | | | | | | | |
| Heeger 2015 | 1.36 [1.05, 1.77] | Z = 2.34 (P = 0.02) | Tau² = 0.05; Chi² = 15.52, df = 3 (P = 0.001); I² = 81% | Heeger2021 | 1.03 [0.87, 1.22] | Z = 0.32 (P = 0.75) | Tau² = 0.03; Chi² = 20.48, df = 4 (P = 0.0004); I² = 80% |
| Iacopino 2020 | 1.78 [1.15, 2.75] | Z = 2.60 (P = 0.009) | Tau² = 0.16; Chi² = 23.71, df = 3 (P < 0.0001); I² = 87% | Iacopino 2020 | 1.04 [0.89, 1.22] | Z = 0.49 (P = 0.62) | Tau² = 0.02; Chi² = 23.19, df = 4 (P = 0.0001); I² = 83% |
| Mugnai 2016 | 1.72 [1.08, 2.73] | Z = 2.31 (P = 0.02) | Tau² = 0.18; Chi² = 19.97, df = 3 (P = 0.0002); I² = 85% | Manfrin 2022 | 1.01 [0.89, 1.16] | Z = 0.20 (P = 0.84) | Tau² = 0.02; Chi² = 16.24, df = 4 (P = 0.003); I² = 75% |
| Pott 2016 | 1.54 [1.09, 2.19] | Z = 2.45 (P = 0.01) | Tau² = 0.09; Chi² = 20.50, df = 3 (P = 0.0001); I² = 85% | Miyazaki 2022 | 1.06 [0.91, 1.25] | Z = 0.76 (P = 0.45) | Tau² = 0.03; Chi² = 20.14, df = 4 (P = 0.0005); I² = 80% |
| Sciarra 2017 | 1.28 [1.05, 1.56] | Z = 2.47 (P = 0.01) | Tau² = 0.02; Chi² = 9.19, df = 3 (P = 0.03); I² = 67% | Moltrasio 2019 | 1.12 [1.00, 1.24] | Z = 2.04 (P = 0.04) | Tau² = 0.01; Chi² = 11.65, df = 4 (P = 0.02); I² = 66% |

**Table S2.** *Continued*

| CB3 vs CB2 | | | | CB4 vs CB2 | | | |
| --- | --- | --- | --- | --- | --- | --- | --- |
| Excluded study | Proportion (95% CI) | Test for overall effect | Heterogeneity | Excluded study | Proportion (95% CI) | Test for overall effect | Heterogeneity |
| LSPV recording | | | | | | | |
|  |  |  |  | Rottner 2020 | 1.07 [0.91, 1.25] | Z = 0.80 (P = 0.42) | Tau² = 0.03; Chi² = 21.54, df = 4 (P = 0.0002); I² = 81% |
| LIPV recording | | | | | | | |
| Heeger 2015 | 1.33 [1.11, 1.60] | Z = 3.09 (P = 0.002) | Tau² = 0.02; Chi² = 5.96, df = 3 (P = 0.11); I² = 50% | Heeger2021 | 1.41 [1.09, 1.83] | Z = 2.64 (P = 0.008) | Tau² = 0.06; Chi² = 18.82, df = 4 (P = 0.0009); I² = 79% |
| Iacopino 2020 | 1.40 [1.19, 1.66] | Z = 4.03 (P < 0.0001) | Tau² = 0.01; Chi² = 4.12, df = 3 (P = 0.25); I² = 27% | Iacopino 2020 | 1.38 [1.12, 1.70] | Z = 2.99 (P = 0.003) | Tau² = 0.04; Chi² = 18.62, df = 4 (P = 0.0009); I² = 79% |
| Mugnai 2016 | 1.40 [1.08, 1.80] | Z = 2.56 (P = 0.01) | Tau² = 0.03; Chi² = 5.95, df = 3 (P = 0.11); I² = 50% | Manfrin 2022 | 1.29 [1.06, 1.57] | Z = 2.53 (P = 0.01) | Tau² = 0.03; Chi² = 15.02, df = 4 (P = 0.005); I² = 73% |
| Pott 2016 | 1.28 [1.10, 1.49] | Z = 3.19 (P = 0.001) | Tau² = 0.01; Chi² = 4.25, df = 3 (P = 0.24); I² = 29% | Miyazaki 2022 | 1.29 [1.07, 1.56] | Z = 2.66 (P = 0.008) | Tau² = 0.03; Chi² = 17.19, df = 4 (P = 0.002); I² = 77% |
| Sciarra 2017 | 1.28 [1.12, 1.47] | Z = 3.64 (P = 0.0003) | Tau² = 0.01; Chi² = 3.98, df = 3 (P = 0.26); I² = 25% | Moltrasio 2019 | 1.21 [1.07, 1.37] | Z = 3.08 (P = 0.002) | Tau² = 0.01; Chi² = 7.65, df = 4 (P = 0.11); I² = 48% |
|  |  |  |  | Rottner 2020 | 1.41 [1.13, 1.76] | Z = 3.04 (P = 0.002) | Tau² = 0.04; Chi² = 15.82, df = 4 (P = 0.003); I² = 75% |
| RSPV recording | | | | | | | |
| Heeger 2015 | 1.33 [0.98, 1.80] | Z = 1.83 (P = 0.07) | Tau² = 0.06; Chi² = 12.74, df = 3 (P = 0.005); I² = 76% | Heeger2021 | 1.40 [1.17, 1.66] | Z = 3.80 (P = 0.0001) | Tau² = 0.02; Chi² = 8.55, df = 4 (P = 0.07); I² = 53% |

**Table S2.** *Continued*

| CB3 vs CB2 | | | | CB4 vs CB2 | | | |
| --- | --- | --- | --- | --- | --- | --- | --- |
| Excluded study | Proportion (95% CI) | Test for overall effect | Heterogeneity | Excluded study | Proportion (95% CI) | Test for overall effect | Heterogeneity |
| RSPV recording | | | | | | | |
| Iacopino 2020 | 1.58 [1.15, 2.18] | Z = 2.81 (P = 0.005) | Tau² = 0.07; Chi² = 10.44, df = 3 (P = 0.02); I² = 71% | Iacopino 2020 | 1.41 [1.20, 1.65] | Z = 4.28 (P < 0.0001) | Tau² = 0.02; Chi² = 9.05, df = 4 (P = 0.06); I² = 56% |
| Mugnai 2016 | 1.52 [0.96, 2.42] | Z = 1.77 (P = 0.08) | Tau² = 0.16; Chi² = 14.11, df = 3 (P = 0.003); I² = 79% | Miyazaki 2022 | 1.40 [1.19, 1.64] | Z = 4.01 (P < 0.0001) | Tau² = 0.02; Chi² = 8.94, df = 4 (P = 0.06); I² = 55% |
| Pott 2016 | 1.42 [0.98, 2.06] | Z = 1.84 (P = 0.07) | Tau² = 0.10; Chi² = 13.74, df = 3 (P = 0.003); I² = 78% | Moltrasio 2019 | 1.36 [1.19, 1.56] | Z = 4.43 (P < 0.00001) | Tau² = 0.01; Chi² = 7.71, df = 4 (P = 0.10); I² = 48% |
| Sciarra 2017 | 1.26 [1.01, 1.57] | Z = 2.01 (P = 0.04) | Tau² = 0.03; Chi² = 8.10, df = 3 (P = 0.04); I² = 63% | Rottner 2020 | 1.34 [1.20, 1.50] | Z = 5.15 (P < 0.00001) | Tau² = 0.00; Chi² = 5.69, df = 4 (P = 0.22); I² = 30% |
|  |  |  |  | Manfrin 2022 | 1.45 [1.30, 1.61] | Z = 6.82 (P < 0.00001) | Tau² = 0.00; Chi² = 3.24, df = 4 (P = 0.52); I² = 0% |
| RIPV recording | | | | | | | |
| Heeger 2015 | 1.35 [0.99, 1.84] | Z = 1.89 (P = 0.06) | Tau² = 0.06; Chi² = 10.13, df = 3 (P = 0.02); I² = 70% | Heeger2021 | 1.49 [1.11, 2.01] | Z = 2.62 (P = 0.009) | Tau² = 0.08; Chi² = 14.70, df = 4 (P = 0.005); I² = 73% |
| Iacopino 2020 | 1.64 [1.21, 2.22] | Z = 3.18 (P = 0.001) | Tau² = 0.05; Chi² = 7.92, df = 3 (P = 0.05); I² = 62% | Iacopino 2020 | 1.39 [1.06, 1.82] | Z = 2.41 (P = 0.02) | Tau² = 0.06; Chi² = 15.73, df = 4 (P = 0.003); I² = 75% |
| Mugnai 2016 | 1.56 [0.96, 2.52] | Z = 1.81 (P = 0.07) | Tau² = 0.17; Chi² = 12.36, df = 3 (P = 0.006); I² = 76% | Manfrin 2022 | 1.32 [1.03, 1.69] | Z = 2.23 (P = 0.03) | Tau² = 0.05; Chi² = 12.13, df = 4 (P = 0.02); I² = 67% |
| Pott 2016 | 1.45 [0.99, 2.12] | Z = 1.90 (P = 0.06) | Tau² = 0.10; Chi² = 11.86, df = 3 (P = 0.008); I² = 75% | Miyazaki 2022 | 1.36 [1.07, 1.72] | Z = 2.51 (P = 0.01) | Tau² = 0.05; Chi² = 15.49, df = 4 (P = 0.004); I² = 74% |

**Table S2.** *Continued*

| CB3 vs CB2 | | | | CB4 vs CB2 | | | |
| --- | --- | --- | --- | --- | --- | --- | --- |
| Excluded study | Proportion (95% CI) | Test for overall effect | Heterogeneity | Excluded study | Proportion (95% CI) | Test for overall effect | Heterogeneity |
| RIPV recording | | | | | | | |
| Sciarra 2017 | 1.33 [1.02, 1.73] | Z = 2.08 (P = 0.04) | Tau² = 0.05; Chi² = 8.73, df = 3 (P = 0.03); I² = 66% | Moltrasio 2019 | 1.25 [1.05, 1.48] | Z = 2.55 (P = 0.01) | Tau² = 0.02; Chi² = 8.05, df = 4 (P = 0.09); I² = 50% |
|  |  |  |  | Rottner 2020 | 1.49 [1.14, 1.94] | Z = 2.96 (P = 0.003) | Tau² = 0.05; Chi² = 12.15, df = 4 (P = 0.02); I² = 67% |
| PVI recording | | | | | | | |
| Fürnkranz 2016 | 1.31 [1.22, 1.40] | Z = 7.88 (P < 0.00001) | Chi² = 29.41, df = 4 (P < 0.00001); I² = 86% | Heeger2021 | 1.46 [1.12, 1.92] | Z = 2.78 (P = 0.005) | Tau² = 0.07; Chi² = 41.57, df = 3 (P < 0.00001); I² = 93% |
| Heeger 2015 | 1.19 [1.12, 1.27] | Z = 5.64 (P < 0.00001) | Chi² = 33.80, df = 4 (P < 0.00001); I² = 88% | Iacopino 2020 | 1.49 [1.20, 1.85] | Z = 3.65 (P = 0.0003) | Tau² = 0.04; Chi² = 23.94, df = 3 (P < 0.0001); I² = 87% |
| Heeger 2019 | 1.25 [1.17, 1.33] | Z = 6.61 (P < 0.00001) | Chi² = 48.06, df = 4 (P < 0.00001); I² = 92% | Manfrin 2022 | 1.40 [1.07, 1.82] | Z = 2.47 (P = 0.01) | Tau² = 0.07; Chi² = 37.02, df = 3 (P < 0.00001); I² = 92% |
| Iacopino 2020 | 1.31 [1.22, 1.40] | Z = 7.76 (P < 0.00001) | Chi² = 37.12, df = 4 (P < 0.00001); I² = 89% | Miyazaki 2022 | 1.42 [1.11, 1.81] | Z = 2.81 (P = 0.005) | Tau² = 0.06; Chi² = 39.69, df = 3 (P < 0.00001); I² = 92% |
| Mugnai 2016 | 1.24 [1.16, 1.34] | Z = 5.89 (P < 0.00001) | Chi² = 47.40, df = 4 (P < 0.00001); I² = 92% | Moltrasio 2019 | 1.26 [1.11, 1.44] | Z = 3.52 (P = 0.0004) | Tau² = 0.01; Chi² = 11.97, df = 3 (P = 0.007); I² = 75% |
| pott 2016 | 1.21 [1.13, 1.29] | Z = 5.60 (P < 0.00001) | Chi² = 38.87, df = 4 (P < 0.00001); I² = 90% |  |  |  |  |

**Table S2.** *Continued*

| CB3 vs CB2 | | | | CB4 vs CB2 | | | |
| --- | --- | --- | --- | --- | --- | --- | --- |
| Excluded study | Proportion (95% CI) | Test for overall effect | Heterogeneity | Excluded study | Proportion (95% CI) | Test for overall effect | Heterogeneity |
| TTI LSPV | | | | | | | |
| Aryana 2016 | 2.21 [-2.40, 6.81] | Z = 0.94 (P = 0.35) | Tau² = 4.87; Chi² = 3.80, df = 3 (P = 0.28); I² = 21% | Heeger2021 | -2.53 [-10.63, 5.56] | Z = 0.61 (P = 0.54) | Tau² = 41.96; Chi² = 7.80, df = 3 (P = 0.05); I² = 62% |
| Heeger 2015 | 2.73 [-0.08, 5.54] | Z = 1.91 (P = 0.06) | Tau² = 0.00; Chi² = 2.34, df = 3 (P = 0.51); I² = 0% | Iacopino 2020 | -1.49 [-8.54, 5.56] | Z = 0.41 (P = 0.68) | Tau² = 31.93; Chi² = 8.21, df = 3 (P = 0.04); I² = 63% |
| Iacopino 2020 | 3.73 [0.79, 6.68] | Z = 2.49 (P = 0.01) | Tau² = 0.00; Chi² = 2.07, df = 3 (P = 0.56); I² = 0% | Manfrin 2022 | 0.24 [-3.92, 4.41] | Z = 0.11 (P = 0.91) | Tau² = 2.65; Chi² = 3.45, df = 3 (P = 0.33); I² = 13% |
| Mugnai 2016 | 3.18 [-1.25, 7.61] | Z = 1.41 (P = 0.16) | Tau² = 5.80; Chi² = 4.10, df = 3 (P = 0.25); I² = 27% | Miyazaki 2022 | -3.38 [-8.40, 1.64] | Z = 1.32 (P = 0.19) | Tau² = 9.25; Chi² = 4.58, df = 3 (P = 0.20); I² = 35% |
| Pott 2016 | 2.63 [-1.27, 6.54] | Z = 1.32 (P = 0.19) | Tau² = 4.46; Chi² = 4.15, df = 3 (P = 0.25); I² = 28% | Moltrasio 2019 | -1.50 [-8.50, 5.50] | Z = 0.42 (P = 0.68) | Tau² = 31.51; Chi² = 8.23, df = 3 (P = 0.04); I² = 64% |
| TTI LIPV | | | | | | | |
| Aryana 2016 | -3.16 [-6.94, 0.62] | Z = 1.64 (P = 0.10) | Tau² = 0.00; Chi² = 0.37, df = 3 (P = 0.95); I² = 0% | Heeger2021 | -3.35 [-6.99, 0.29] | Z = 1.80 (P = 0.07) | Tau² = 0.00; Chi² = 0.48, df = 3 (P = 0.92); I² = 0% |
| Heeger 2015 | -0.93 [-4.40, 2.54] | Z = 0.53 (P = 0.60) | Tau² = 3.34; Chi² = 4.06, df = 3 (P = 0.26); I² = 26% | Iacopino 2020 | -4.16 [-7.11, -1.21] | Z = 2.76 (P = 0.006) | Tau² = 0.00; Chi² = 1.04, df = 3 (P = 0.79); I² = 0% |
| Iacopino 2020 | -0.41 [-3.51, 2.69] | Z = 0.26 (P = 0.80) | Tau² = 1.44; Chi² = 3.42, df = 3 (P = 0.33); I² = 12% | Manfrin 2022 | -4.53 [-7.58, -1.48] | Z = 2.91 (P = 0.004) | Tau² = 0.00; Chi² = 1.01, df = 3 (P = 0.80); I² = 0% |
| Mugnai 2016 | 0.61 [-2.60, 3.83] | Z = 0.37 (P = 0.71) | Tau² = 0.00; Chi² = 2.80, df = 3 (P = 0.42); I² = 0% | Miyazaki 2022 | -4.75 [-8.20, -1.30] | Z = 2.70 (P = 0.007) | Tau² = 0.00; Chi² = 0.97, df = 3 (P = 0.81); I² = 0% |

**Table S2.** *Continued*

| CB3 vs CB2 | | | | CB4 vs CB2 | | | |
| --- | --- | --- | --- | --- | --- | --- | --- |
| Excluded study | Proportion (95% CI) | Test for overall effect | Heterogeneity | Excluded study | Proportion (95% CI) | Test for overall effect | Heterogeneity |
| TTI LIPV | | | | | | | |
| Pott 2016 | -0.35 [-3.30, 2.60] | Z = 0.23 (P = 0.82) | Tau² = 0.94; Chi² = 3.29, df = 3 (P = 0.35); I² = 9% | Moltrasio 2019 | -4.56 [-7.51, -1.62] | Z = 3.04 (P = 0.002) | Tau² = 0.00; Chi² = 0.76, df = 3 (P = 0.86); I² = 0% |
| TTI RSPV | | | | | | | |
| Aryana 2016 | -2.57 [-9.32, 4.18] | -2.57 [-9.32, 4.18] | Tau² = 21.43; Chi² = 5.58, df = 3 (P = 0.13); I² = 46% | Heeger2021 | -1.95 [-5.52, 1.62] | Z = 1.07 (P = 0.28) | Tau² = 0.00; Chi² = 0.89, df = 3 (P = 0.83); I² = 0% |
| Heeger 2015 | -1.22 [-5.87, 3.44] | Z = 0.51 (P = 0.61) | Tau² = 11.62; Chi² = 7.29, df = 3 (P = 0.06); I² = 59% | Iacopino 2020 | 0.34 [-5.44, 6.12] | Z = 0.11 (P = 0.91) | Tau² = 18.98; Chi² = 7.09, df = 3 (P = 0.07); I² = 58% |
| Iacopino 2020 | -2.01 [-6.45, 2.42] | Z = 0.89 (P = 0.37) | Tau² = 8.68; Chi² = 5.92, df = 3 (P = 0.12); I² = 49% | Manfrin 2022 | 1.20 [-3.88, 6.28] | Z = 0.46 (P = 0.64) | Tau² = 11.71; Chi² = 5.46, df = 3 (P = 0.14); I² = 45% |
| Mugnai 2016 | -1.22 [-8.43, 5.99] | Z = 0.33 (P = 0.74) | Tau² = 27.29; Chi² = 6.40, df = 3 (P = 0.09); I² = 53% | Miyazaki 2022 | 0.00 [-6.81, 6.81] | Z = 0.00 (P = 1.00) | Tau² = 27.08; Chi² = 7.11, df = 3 (P = 0.07); I² = 58% |
| Pott 2016 | -0.04 [-2.54, 2.45] | Z = 0.03 (P = 0.97) | Tau² = 0.00; Chi² = 2.49, df = 3 (P = 0.48); I² = 0% | Moltrasio 2019 | 0.31 [-4.93, 5.55] | Z = 0.12 (P = 0.91) | Tau² = 16.20; Chi² = 7.10, df = 3 (P = 0.07); I² = 58% |
| TTI RIPV | | | | | | | |
| Aryana 2016 | 1.40 [-3.95, 6.76] | Z = 0.51 (P = 0.61) | Tau² = 9.84; Chi² = 4.29, df = 3 (P = 0.23); I² = 30% | Heeger2021 | -9.23 [-22.01, 3.55] | Z = 1.42 (P = 0.16) | Tau² = 141.24; Chi² = 19.59, df = 3 (P = 0.0002); I² = 85% |
| Heeger 2015 | 2.18 [-1.45, 5.82] | Z = 1.18 (P = 0.24) | Tau² = 4.30; Chi² = 4.41, df = 3 (P = 0.22); I² = 32% | Iacopino 2020 | -5.74 [-21.09, 9.60] | Z = 0.73 (P = 0.46) | Tau² = 222.25; Chi² = 38.59, df = 3 (P < 0.00001); I² = 92% |

**Table S2.** *Continued*

| CB3 vs CB2 | | | | CB4 vs CB2 | | | |
| --- | --- | --- | --- | --- | --- | --- | --- |
| Excluded study | Proportion (95% CI) | Test for overall effect | Heterogeneity | Excluded study | Proportion (95% CI) | Test for overall effect | Heterogeneity |
| TTI RIPV | | | | | | | |
| Iacopino 2020 | 3.09 [0.41, 5.76] | Z = 2.26 (P = 0.02) | Tau² = 0.00; Chi² = 0.50, df = 3 (P = 0.92); I² = 0% | Miyazaki 2022 | -1.80 [-13.64, 10.05] | Z = 0.30 (P = 0.77) | Tau² = 117.94; Chi² = 17.29, df = 3 (P = 0.0006); I² = 83% |
| Mugnai 2016 | 1.48 [-3.98, 6.93] | Z = 0.53 (P = 0.60) | Tau² = 10.45; Chi² = 4.36, df = 3 (P = 0.23); I² = 31% | Moltrasio 2019 | -8.27 [-23.67, 7.13] | Z = 1.05 (P = 0.29) | Tau² = 220.23; Chi² = 33.87, df = 3 (P < 0.00001); I² = 91% |
| Pott 2016 | 1.92 [-1.38, 5.22] | Z = 1.14 (P = 0.25) | Tau² = 2.47; Chi² = 3.78, df = 3 (P = 0.29); I² = 21% | Manfrin 2022 | -1.78 [-15.21, 11.64] | Z = 0.26 (P = 0.79) | Tau² = 167.51; Chi² = 31.01, df = 3 (P < 0.00001); I² = 90% |
| Total TTI | | | | | | | |
| Aryana 2016 | 0.41 [-3.63, 4.44] | Z = 0.20 (P = 0.84) | Tau² = 4.75; Chi² = 4.16, df = 3 (P = 0.24); I² = 28% | Not applicable |  |  |  |
| Fürnkranz 2016 | 1.04 [-0.67, 2.74] | Z = 1.19 (P = 0.23) | Tau² = 0.00; Chi² = 2.88, df = 3 (P = 0.41); I² = 0% |  |  |  |  |
| Heeger 2015 | 0.64 [-1.06, 2.34] | Z = 0.74 (P = 0.46) | Tau² = 0.00; Chi² = 2.95, df = 3 (P = 0.40); I² = 0% |  |  |  |  |
| Heeger 2019 | 0.35 [-2.26, 2.96] | Z = 0.26 (P = 0.80) | Tau² = 1.80; Chi² = 3.74, df = 3 (P = 0.29); I² = 20% |  |  |  |  |
| Pott 2016 | 1.14 [-0.71, 2.99] | Z = 1.21 (P = 0.23) | Tau² = 0.13; Chi² = 3.05, df = 3 (P = 0.38); I² = 2% |  |  |  |  |

**Table S2.** *Continued*

| CB3 vs CB2 | | | | CB4 vs CB2 | | | |
| --- | --- | --- | --- | --- | --- | --- | --- |
| Excluded study | Proportion (95% CI) | Test for overall effect | Heterogeneity | Excluded study | Proportion (95% CI) | Test for overall effect | Heterogeneity |
| Mean nadir temperature LSPV | | | | | | | |
| Aryana 2016 | 3.72 [0.17, 7.28] | Z = 2.05 (P = 0.04) | Tau² = 14.99; Chi² = 51.66, df = 4 (P < 0.00001); I² = 92% | Iacopino 2020 | -2.96 [-5.68, -0.23] | Z = 2.13 (P = 0.03) | Tau² = 4.41; Chi² = 8.53, df = 2 (P = 0.01); I² = 77% |
| Fürnkranz 2016 | 3.40 [-0.32, 7.13] | Z = 1.79 (P = 0.07) | Tau² = 16.48; Chi² = 54.79, df = 4 (P < 0.00001); I² = 93% | Manfrin 2022 | -1.28 [-3.75, 1.20] | Z = 1.01 (P = 0.31) | Tau² = 3.80; Chi² = 10.23, df = 2 (P = 0.006); I² = 80% |
| Heeger 2015 | 3.27 [-0.47, 7.00] | Z = 1.71 (P = 0.09) | Tau² = 16.62; Chi² = 55.46, df = 4 (P < 0.00001); I² = 93% | Miyazaki 2022 | -2.64 [-6.17, 0.88] | Z = 1.47 (P = 0.14) | Tau² = 8.43; Chi² = 16.38, df = 2 (P = 0.0003); I² = 88% |
| Iacopino 2020 | 1.44 [0.48, 2.40] | Z = 2.95 (P = 0.003) | Chi² = 3.64, df = 4 (P = 0.46); I² = 0% | Moltrasio 2019 | -1.34 [-3.90, 1.22] | Z = 1.03 (P = 0.30) | Tau² = 4.09; Chi² = 10.50, df = 2 (P = 0.005); I² = 81% |
| Mugnai 2016 | 3.58 [-0.25, 7.41] | Z = 1.83 (P = 0.07) | Tau² = 17.23; Chi² = 42.13, df = 4 (P < 0.00001); I² = 91% |  |  |  |  |
| Pott 2016 | 3.09 [-0.65, 6.83] | Z = 1.62 (P = 0.11) | Tau² = 16.68; Chi² = 55.47, df = 4 (P < 0.00001); I² = 93% |  |  |  |  |
| Mean nadir temperature LIPV | | | | | | | |
| Aryana 2016 | 4.29 [1.51, 7.06] | Z = 3.03 (P = 0.002) | Tau² = 8.88; Chi² = 43.59, df = 4 (P < 0.00001); I² = 91% | Iacopino 2020 | -2.96 [-5.68, -0.23] | Z = 2.13 (P = 0.03) | Tau² = 4.41; Chi² = 8.53, df = 2 (P = 0.01); I² = 77% |
| Fürnkranz 2016 | 4.12 [1.08, 7.17] | Z = 2.65 (P = 0.008) | Tau² = 10.86; Chi² = 45.89, df = 4 (P < 0.00001); I² = 91% | Manfrin 2022 | -1.28 [-3.75, 1.20] | Z = 1.01 (P = 0.31) | Tau² = 3.80; Chi² = 10.23, df = 2 (P = 0.006); I² = 80% |
| Heeger 2015 | 3.35 [0.82, 5.89] | Z = 2.59 (P = 0.010) | Tau² = 7.43; Chi² = 41.41, df = 4 (P < 0.00001); I² = 90% | Miyazaki 2022 | -2.60 [-6.02, 0.83] | Z = 1.48 (P = 0.14) | Tau² = 8.20; Chi² = 19.56, df = 2 (P < 0.0001); I² = 90% |

**Table S2.** *Continued*

| CB3 vs CB2 | | | | CB4 vs CB2 | | | |
| --- | --- | --- | --- | --- | --- | --- | --- |
| Excluded study | Proportion (95% CI) | Test for overall effect | Heterogeneity | Excluded study | Proportion (95% CI) | Test for overall effect | Heterogeneity |
| Mean nadir temperature LIPV | | | | | | | |
| Iacopino 2020 | 2.49 [0.90, 4.08] | Z = 3.07 (P = 0.002) | Tau² = 2.35; Chi² = 15.55, df = 4 (P = 0.004); I² = 74% | Moltrasio 2019 | -2.64 [-6.17, 0.88] | Z = 1.47 (P = 0.14) | Tau² = 8.43; Chi² = 16.38, df = 2 (P = 0.0003); I² = 88% |
| Mugnai 2016 | 4.37 [1.61, 7.12] | Z = 3.10 (P = 0.002) | Tau² = 8.63; Chi² = 35.09, df = 4 (P < 0.00001); I² = 89% |  |  |  |  |
| Pott 2016 | 3.61 [0.91, 6.31] | Z = 2.62 (P = 0.009) | Tau² = 8.44; Chi² = 44.14, df = 4 (P < 0.00001); I² = 91% |  |  |  |  |
| Mean nadir temperature RSPV | | | | | | | |
| Aryana 2016 | 4.86 [1.90, 7.82] | Z = 3.22 (P = 0.001) | Tau² = 9.81; Chi² = 33.17, df = 4 (P < 0.00001); I² = 88% | Iacopino 2020 | -3.00 [-5.48, -0.53] | Z = 2.38 (P = 0.02) | Tau² = 3.74; Chi² = 9.24, df = 2 (P = 0.010); I² = 78% |
| Fürnkranz 2016 | 4.15 [1.03, 7.28] | Z = 2.61 (P = 0.009) | Tau² = 11.24; Chi² = 44.77, df = 4 (P < 0.00001); I² = 91% | Manfrin 2022 | -1.48 [-4.23, 1.27] | Z = 1.06 (P = 0.29) | Tau² = 5.07; Chi² = 14.54, df = 2 (P = 0.0007); I² = 86% |
| Heeger 2015 | 3.91 [0.94, 6.87] | Z = 2.58 (P = 0.010) | Tau² = 10.16; Chi² = 43.51, df = 4 (P < 0.00001); I² = 91% | Miyazaki 2022 | -2.60 [-6.02, 0.83] | Z = 1.48 (P = 0.14) | Tau² = 8.20; Chi² = 19.56, df = 2 (P < 0.0001); I² = 90% |
| Iacopino 2020 | 2.80 [0.80, 4.80] | Z = 2.75 (P = 0.006) | Tau² = 3.90; Chi² = 18.83, df = 4 (P = 0.0008); I² = 79% | Moltrasio 2019 | -1.30 [-3.76, 1.16] | Z = 1.04 (P = 0.30) | Tau² = 3.88; Chi² = 11.55, df = 2 (P = 0.003); I² = 83% |
| Mugnai 2016 | 4.61 [1.19, 8.03] | Z = 2.64 (P = 0.008) | Tau² = 13.53; Chi² = 38.98, df = 4 (P < 0.00001); I² = 90% |  |  |  |  |
| Pott 2016 | 3.58 [0.75, 6.41] | Z = 2.48 (P = 0.01) | Tau² = 9.05; Chi² = 38.11, df = 4 (P < 0.00001); I² = 90% |  |  |  |  |

**Table S2.** *Continued*

| CB3 vs CB2 | | | | CB4 vs CB2 | | | |
| --- | --- | --- | --- | --- | --- | --- | --- |
| Excluded study | Proportion (95% CI) | Test for overall effect | Heterogeneity | Excluded study | Proportion (95% CI) | Test for overall effect | Heterogeneity |
| Mean nadir temperature RIPV | | | | | | | |
| Aryana 2016 | 3.78 [0.96, 6.61] | Z = 2.62 (P = 0.009) | Tau² = 8.90; Chi² = 31.35, df = 4 (P < 0.00001); I² = 87% | Iacopino 2020 | -3.00 [-5.48, -0.53] | Z = 2.38 (P = 0.02) | Tau² = 3.74; Chi² = 9.24, df = 2 (P = 0.010); I² = 78% |
| Fürnkranz 2016 | 3.60 [0.60, 6.59] | Z = 2.35 (P = 0.02) | Tau² = 10.28; Chi² = 39.16, df = 4 (P < 0.00001); I² = 90% | Manfrin 2022 | -1.48 [-4.23, 1.27] | Z = 1.06 (P = 0.29) | Tau² = 5.07; Chi² = 14.54, df = 2 (P = 0.0007); I² = 86% |
| Heeger 2015 | 3.18 [0.27, 6.09] | Z = 2.14 (P = 0.03) | Tau² = 9.83; Chi² = 41.87, df = 4 (P < 0.00001); I² = 90% | Miyazaki 2022 | -2.60 [-6.02, 0.83] | Z = 1.48 (P = 0.14) | Tau² = 8.20; Chi² = 19.56, df = 2 (P < 0.0001); I² = 90% |
| Iacopino 2020 | 1.84 [0.27, 3.41] | Z = 2.29 (P = 0.02) | Tau² = 1.97; Chi² = 11.05, df = 4 (P = 0.03); I² = 64% | Moltrasio 2019 | -1.30 [-3.76, 1.16] | Z = 1.04 (P = 0.30) | Tau² = 3.88; Chi² = 11.55, df = 2 (P = 0.003); I² = 83% |
| Mugnai 2016 | 3.44 [0.16, 6.72] | Tau² = 12.49; Chi² = 40.77, df = 4 (P < 0.00001); I² = 90% | Tau² = 12.49; Chi² = 40.77, df = 4 (P < 0.00001); I² = 90% |  |  |  |  |
| Pott 2016 | 2.70 [-0.07, 5.46] | Z = 1.91 (P = 0.06) | Tau² = 8.73; Chi² = 37.66, df = 4 (P < 0.00001); I² = 89% |  |  |  |  |
| Mean nadir temperature overall | | | | | | | |
| Aryana 2016 | 3.56 [2.67, 4.45] | Z = 7.83 (P < 0.00001) | Tau² = 0.35; Chi² = 6.18, df = 4 (P = 0.19); I² = 35% | Not applicable |  |  |  |
| Fürnkranz 2016 | 3.07 [1.28, 4.85] | Z = 3.36 (P = 0.0008) | Tau² = 3.51; Chi² = 36.65, df = 4 (P < 0.00001); I² = 89% |  |  |  |  |

**Table S2.** *Continued*

| CB3 vs CB2 | | | | CB4 vs CB2 | | | |
| --- | --- | --- | --- | --- | --- | --- | --- |
| Excluded study | Proportion (95% CI) | Test for overall effect | Heterogeneity | Excluded study | Proportion (95% CI) | Test for overall effect | Heterogeneity |
| Mean nadir temperature overall | | | | | | | |
| Heeger 2015 | 2.68 [0.88, 4.48] | Z = 2.91 (P = 0.004) | Tau² = 3.45; Chi² = 31.76, df = 4 (P < 0.00001); I² = 87% |  |  |  |  |
| Heeger 2019 | 2.88 [0.87, 4.89] | Z = 2.80 (P = 0.005) | Tau² = 4.42; Chi² = 35.46, df = 4 (P < 0.00001); I² = 89% |  |  |  |  |
| Pott 2016 | 2.48 [0.91, 4.04] | Z = 3.10 (P = 0.002) | Tau² = 2.42; Chi² = 22.89, df = 4 (P = 0.0001); I² = 83% |  |  |  |  |
| Sciarra 2017 | 2.80 [1.08, 4.51] | Z = 3.19 (P = 0.001) | Tau² = 3.32; Chi² = 36.22, df = 4 (P < 0.00001); I² = 89% |  |  |  |  |
| AF recrudescence | | | | | | | |
| Aryana 2016 | 0.88 [0.62, 1.26] | Z = 0.69 (P = 0.49) | Tau² = 0.00; Chi² = 3.06, df = 3 (P = 0.38); I² = 2% | Not applicable |  |  |  |
| Heeger 2019 | 0.92 [0.64, 1.33] | Z = 0.45 (P = 0.65) | Tau² = 0.02; Chi² = 3.48, df = 3 (P = 0.32); I² = 14% |  |  |  |  |
| Mugnai 2016 | Z = 0.65 (P = 0.52) | Z = 0.65 (P = 0.52) | Tau² = 0.00; Chi² = 0.29, df = 3 (P = 0.96); I² = 0% |  |  |  |  |
| Pott 2016 | 0.93 [0.61, 1.43] | Z = 0.33 (P = 0.74) | Tau² = 0.03; Chi² = 3.63, df = 3 (P = 0.30); I² = 17% |  |  |  |  |
| Sciarra 2017 | 0.88 [0.62, 1.26] | Z = 0.69 (P = 0.49) | Tau² = 0.00; Chi² = 3.06, df = 3 (P = 0.38); I² = 2% |  |  |  |  |

**Table S2.** *Continued*

| CB3 vs CB2 | | | | CB4 vs CB2 | | | |
| --- | --- | --- | --- | --- | --- | --- | --- |
| Excluded study | Proportion (95% CI) | Test for overall effect | Heterogeneity | Excluded study | Proportion (95% CI) | Test for overall effect | Heterogeneity |
| Pericardial effusion / cardiac tamponade | | | | | | | |
| Not applicable |  |  |  |  |  |  |  |
| PNP | | | | | | | |
| Aryana 2016 | 0.68 [0.25, 1.85] | Z = 0.76 (P = 0.45) | Tau² = 0.00; Chi² = 2.25, df = 4 (P = 0.69); I² = 0% | Heeger2021 | 0.99 [0.41, 2.41] | Z = 0.02 (P = 0.99) | Tau² = 0.00; Chi² = 2.52, df = 4 (P = 0.64); I² = 0% |
| Fürnkranz 2016 | 0.79 [0.29, 2.17] | Z = 0.45 (P = 0.65) | Tau² = 0.00; Chi² = 2.27, df = 4 (P = 0.69); I² = 0% | Iacopino 2020 | 0.99 [0.44, 2.23] | Z = 0.02 (P = 0.99) | Tau² = 0.00; Chi² = 2.52, df = 4 (P = 0.64); I² = 0% |
| Heeger 2015 | 0.64 [0.23, 1.74] | Z = 0.88 (P = 0.38) | Tau² = 0.00; Chi² = 1.69, df = 4 (P = 0.79); I² = 0% | Manfrin 2022 | 1.23 [0.49, 3.11] | Z = 0.44 (P = 0.66) | Tau² = 0.00; Chi² = 1.83, df = 4 (P = 0.77); I² = 0% |
| Heeger 2019 | 0.63 [0.23, 1.74] | Z = 0.88 (P = 0.38) | Tau² = 0.00; Chi² = 1.68, df = 4 (P = 0.79); I² = 0% | Miyazaki 2022 | 0.90 [0.40, 2.00] | Z = 0.27 (P = 0.79) | Tau² = 0.00; Chi² = 1.56, df = 4 (P = 0.82); I² = 0% |
| Iacopino 2020 | 0.89 [0.30, 2.66] | Z = 0.21 (P = 0.83) | Tau² = 0.00; Chi² = 2.02, df = 4 (P = 0.73); I² = 0% | Moltrasio 2019 | 1.10 [0.46, 2.61] | Z = 0.21 (P = 0.83) | Tau² = 0.00; Chi² = 2.26, df = 4 (P = 0.69); I² = 0% |
| Mugnai 2016 | 0.89 [0.26, 3.07] | Z = 0.19 (P = 0.85) | Tau² = 0.00; Chi² = 2.31, df = 4 (P = 0.68); I² = 0% | Rottner 2020 | 0.86 [0.37, 1.96] | Z = 0.37 (P = 0.71) | Tau² = 0.00; Chi² = 1.46, df = 4 (P = 0.83); I² = 0% |

Sensitivity analyses were not applicable for studies with only two trials.
